# Proton Conductivities of Stepwise Protonated Imidazole‐Fused Tetraphenylene Derivatives

**DOI:** 10.1002/chem.202502622

**Published:** 2025-10-09

**Authors:** Mu Li, Takashi Takeda, Shun Dekura, Tetsu Sato, Tomoyuki Akutagawa

**Affiliations:** ^1^ Graduate School of Engineering Tohoku University Sendai 980–8579 Japan; ^2^ Institute of Multidisciplinary Research for Advanced Materials (IMRAM) Tohoku University 2‐1‐1 Katahira, Aoba‐ku Sendai 980–8577 Japan; ^3^ Faculty of Science Shinshu University 3‐1‐1 Asahi Matsumoto 390–8621 Japan

**Keywords:** conductivity switching, H_2_O sorption, hydrogen bonds, proton conductivity, tetraphenylene

## Abstract

The control of crystal structure and proton conductivity was evaluated by stepwise protonation of tetraphenylene molecules (**ImTP**) containing a imidazole group. By controlling the degree of protonation from neutral **ImTP** to **H_1_ImTP⁺**, **H_2_ImTP^2^⁺**, and **H_4_ImTP⁴⁺**, we demonstrated that the balance between intermolecular hydrogen bonding and electrostatic interactions affects structural stability and proton conductivity. X‐ray diffraction analysis revealed that neutral **ImTP** forms a 2D hydrogen bond network, while **H_1_ImTP⁺** exhibits enhanced structural stability due to N─H⁺···Cl^−^ electrostatic interactions. **H_2_ImTP^2^⁺** forms a 3D network, and **H_4_ImTP⁴⁺** becomes amorphous. Under 40% relative humidity conditions, these compounds adsorb H_2_O to form proton conduction pathways, exhibiting conductivities of 3.20 × 10^−^^7^, 3.38 × 10^−^^6^, 1.35 × 10^−^^6^, and 6.20 × 10^−^^6^ S cm^−^^1^ at ∼363 K for *n* = 0, 1, 2, and 4 of **H*_n_* ImTP*^n^* ⁺**Cl^−^ *_n_* system, respectively. In particular, **H_1_ImTP⁺** exhibits an optimal balance between hydrogen bonding and electrostatic interactions, resulting in high structural stability, reversible H_2_O adsorption, and proton conductivity.

## Graphical abstract

Proton conducting tetraphenylene molecules (**ImTP**) containing a imidazole group were controlled by the degree of protonation. By controlling the degree of protonation from neutral **ImTP** to **H_1_ImTP⁺**, **H_2_ImTP^2^⁺**, and **H_4_ImTP⁴⁺**, we demonstrated that the balance between intermolecular hydrogen bonding and electrostatic interactions affects structural stability and proton conductivity.

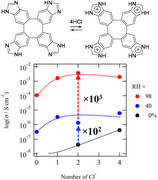

## 1 Introduction

Proton‐conducting materials play a central role in next‐generation energy devices such as fuel cells, electrochemical sensors, and humidity sensors, and are essential for the realization of highly efficient and environmentally friendly energy conversion and storage systems.^[^ ^1^, ^2^, ^3^, ^4^, ^5^ ^]^ In particular, the development of high‐performance proton conductors is an urgent issue in the construction of a sustainable energy society.^[^ ^6^, ^7^ ^]^ Conventional proton conductors have primarily relied on inorganic solid electrolytes such as zirconium phosphate and polymer electrolyte membranes like Nafion.^[^ ^8^, ^9^ ^]^ However, these materials face challenges such as dependence on high‐humidity environments, stability at high temperatures, manufacturing costs, and environmental impact. Therefore, there is a strong demand for the development of new proton conductive materials that can function stably over a wide temperature range and control their properties according to humidity conditions.

In recent years, molecular crystals have attracted attention as materials capable of precise design of proton conduction pathways due to their structural flexibility and chemical diversity.^[^ ^10^, ^11^, ^12^, ^13^, ^14^, ^15^, ^16^, ^17^, ^18^ ^]^ In particular, organic molecules, which allow precise control of intermolecular interactions, are attractive from the perspective of functional material design using a crystallographic approach. Furthermore, the ability to systematically control the protonation state of molecules in a stepwise manner to systematically alter crystal structures and properties provides a powerful tool for understanding structure property correlations.^[^ ^10^, ^19^, ^20^ ^]^ Changes in the charge state of molecules due to protonation can influence crystal structures through alterations in hydrogen bonding patterns, the introduction of electrostatic interactions, and the reorganization of molecular arrangements, potentially leading to dramatic changes in properties.

From the perspective of intermolecular interactions, hydrogen bonds are directional and play an important role in the formation of crystal structures.^[^ ^16^, ^21^, ^22^ ^]^ They are relatively weak interactions and therefore respond flexibly to environmental changes. In contrast, electrostatic interactions are stronger interactions, while their isotropic nature makes it difficult to predict crystal structures. By controlling protonation degree, it is possible to gradually adjust the balance between these two interactions, enabling the design of materials that combine structural stability and functional flexibility. In particular, structural responsiveness to the adsorption and desorption of guest molecules such as H_2_O, and the formation and control of proton conduction pathways associated with this, are important issues in the development of next‐generation proton‐conducting materials.^[^ ^23^, ^24^, ^25^, ^26^, ^27^ ^]^

For instance, it has been shown that the dimensionality of the hydrogen bonding network formed by the counter anion H_2_PO_4_^−^ (e.g., 1D chains, 2D sheets, or 3D networks) significantly influences proton conductivity in various combinations of haloanilinium (X‐Ani^+^) salts and dihydrogen phosphate anions (H_2_PO_4_^−^).^[^ ^28^ ^]^ In particular, it has been reported that a uniform 3D hydrogen bonding network achieves high conductivity. Furthermore, in hydrogen‐bonding organic acid‐base salts, cation‐anion salts between the planar molecules of 2,2′‐diaminobis(tetrazolium) (DABT) derivatives and H_3_PO_4_ exhibit proton conductivity exceeding 10^−^^4^ S cm^−^^1^ and anisotropic conductivity characteristics.^[^ ^29^ ^]^ By controlling crystallization conditions, divalent cationic H_2_DABT^2^⁺(H_2_PO_4_^−^)_2_ salts or mixed protonic (H_2_DABT^2^⁺)(H_2_PO_4_^−^)_2_(H_3_PO_4_)_2_ salts with different 1D, 2D, and 3D hydrogen bonding networks can be formed. The structural isomers 2,2′‐diamino‐4,4′‐bithiazolium (2,4‐DABT) and 2,2′‐diamino‐5,5′‐bithiazolium (2,5‐DABT) form (H_2_DABT^2^⁺)(H_2_PO_4_^−^)_2_ and/or (H_2_DABT^2^⁺)(H_2_PO_4_^−^)_2_(H_3_PO_4_)_2_ salts with the same stoichiometry, but their packing structures are significantly different. In the latter salt, H_2_PO_4_^−^ and H_3_PO_4_, which are different protonated species in the hydrogen bonding network, coexist in the crystal. Four and ten protons (H^+^: carrier concentration) per H_2_DABT^2^⁺ molecule are present in the (H_2_DABT^2^⁺)(H_2_PO_4_^−^)_2_ and (H_2_DABT^2^⁺)(H_2_PO_4_^−^)_2_(H_3_PO_4_)_2_ salts, respectively, which is the reason for the high proton conductivity in the mixed protonated crystal of the latter. To design anhydrous proton conductors, both mixed proton transfer states and uniform O─H···O hydrogen bonding interactions must be considered as essential elements.

Compared to simple π‐planar cations such as Ani^+^ and H_2_DABT^2+^, nonplanar π‐conjugated molecules form more complex 3D network structures, which may contribute to improved proton conductivity. For example, tetraphenylene (**TP**) derivatives, which are nonplanar π‐molecules, enable precise design of molecular packing and intermolecular interactions due to their curved, saddle‐shaped structure and abundant chemical modification sites, and are attracting attention from the perspective of electronic structure and physical property control.^[^ ^30^, ^31^ ^]^ Among these, octaaminotetraphenylene (**OATP**), which has eight amino groups, forms a stable tetracation (**H_4_OATP^4^⁺**) in its fully protonated state due to its characteristic nonplanar structure.^[^ ^32^ ^]^ When combined with H_2_PO_4_^−^ as the counter anion, it constructs a robust hydrogen bonding network and exhibits proton conductivity. In fact, in hydrogen‐bonded **OATP** salts, proton conductivity ON/OFF switching phenomena have been confirmed through crystal amorphous transitions associated with H_2_O adsorption and desorption, with proton conductivity increasing by two orders of magnitude after H_2_O adsorption. This is attributed to the transformation of the amorphous salt in the anhydrous state into a crystalline state upon water absorption, thereby forming an efficient proton conduction pathway.

Various molecules are used as molecular proton conductors.^[^ ^1^, ^11^, ^15^, ^17^, ^33^ ^]^ Among them, imidazole (**Im**) is a five‐membered heterocyclic ring containing nitrogen atoms.^[^ ^13^, ^14^, ^20^, ^34^, ^35^ ^]^ It possesses both proton donor and proton acceptor properties, enabling proton relay transport based on the Grotthuss mechanism.^[^ ^5^, ^10^, ^20^, ^36^ ^]^ As a result, it has garnered attention as a proton conductor capable of operating under nonhumidified conditions without water. Applications of **Im** crystals or **Im** units incorporated into polymer main chains or side chains in polymer electrolytes, as well as coordination polymers (MOFs) containing **Im** groups, have been reported, achieving proton conductivities exceeding 10^−^^3^ S cm^−^^1^.^[^ ^37^, ^38^, ^39^ ^]^ Furthermore, development of proton conductors using triazole, which increases the number of protonation sites compared to **Im**, is also underway.^[^ ^10^, ^40^ ^]^

**Scheme 1:**
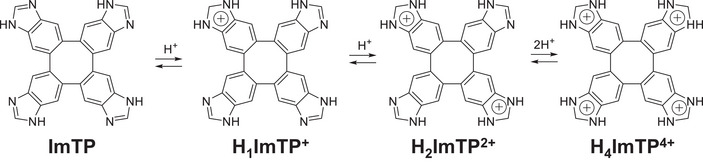
Chemical structures and proton‐transfer processes from **ImTP**, **H_1_ImTP** ^+^, **H_2_ImTP** ^2+^, to **H_4_ImTP** ^4+^.

In this study, we aim to develop a new proton conductor that maximizes the characteristics of the **Im** unit, and we are working to precisely control the molecular arrangement and elucidate the conduction mechanism. We focused on the **ImTP** molecule, in which four **Im** units are fused to a **TP** core, and investigated the changes in intermolecular interactions and the stabilization mechanism of the crystal structure through stepwise protonation. The conjugate acid of benzimidazole has a p*K*_a_ of approximately 5.5,^[^ ^41^ ^]^ enabling stepwise protonation through acid addition. Utilizing this property, we synthesized a series of compounds with precisely controlled protonation degrees (n = 0, 1, 2, 4) from neutral **ImTP** to monoprotonated **H_1_ImTP⁺**, diprotonated **H_2_ImTP^2^⁺**, and tetraprotonated **H_4_ImTP^4^⁺** (Scheme 1), and evaluated their crystal structures, hydrogen‐bonding networks, guest molecule adsorption/desorption characteristics, and proton conductivities in detail. As the degree of protonation increases, changes in crystal symmetry and intermolecular interaction networks are expected to directly influence water molecule adsorption/desorption properties and proton conductivity. This study aims to systematically elucidate the effects of intermolecular interaction balancing through stepwise protonation control on crystal structural stability and functional properties.

## 2 Results and Discussion

### 2.1 Synthesis and the Guest Adsorption State

**ImTP** was newly prepared by condensation of **OATP** with triethyl orthoformate (see the Experimental Section for detail). The p*K*_a_ of benzimidazolium is approximately 5.5, and the four benzimidazole units in neutral **ImTP** molecule can be sequentially protonated to afforded **H_1_ImTP^+^**, **H_2_ImTP^2+^**, and **H_4_ImTP^4+^**. Consistent with this, comparative ^1^H NMR spectra in DMSO‐*d*_6_ of isolated **ImTP**, **H_1_ImTP^+^**, **H_2_ImTP^2+^**, and **H_4_ImTP^4+^** showed progressive downfield shifts with increasing protonation (Figures S1, S2). The H_a_ signal of the benzene ring moved from *δ* = 7.25–7.36 ppm to 7.43, 7.51, and 7.62 ppm, and the H_b_ proton of the imidazole shifted from *δ* = 8.16 ppm to 8.58, 8.84, and 9.20 ppm. In solution, the stepwise protonation of **ImTP** due to the stepwise addition of HCl was confirmed.

Table 1 summarizes the compositions of as‐grown crystals containing **ImTP**, **H_1_ImTP^+^**, **H_2_ImTP^2+^**, and **H_4_ImTP^4+^**, as well as the compositions determined from elemental analysis and TG (thermogravimetry) measurements of samples stored under atmospheric conditions after vacuum drying. Neutral **ImTP** was isolated as a single crystal sample with the composition **ImTP**•(Et_2_O)•3(H_2_O), which includes the solvent (guest) molecules Et_2_O and H_2_O. Among the protonated salts, **H_1_ImTP^+^** Cl^−^•2(CHCl_3_) and **H_2_ImTP^2+^** Cl^−^_2_•4(THF) were isolated as single crystals. On the other hand, although single crystals could not be prepared, **H_4_ImTP^4^⁺**Cl^−^_4_•6(H_2_O) was obtained as a powder sample. **H_3_ImTP^3^⁺**Cl^−^_3_ was obtained as a mixture of protonated salts, and isolation as a single composition was difficult. The single crystals of **ImTP**•(Et_2_O)•3(H_2_O), **H_1_ImTP⁺**Cl^−^•2(CHCl_3_), and **H_2_ImTP^2^⁺**Cl^−^_2_•4(THF) were analyzed by single‐crystal X‐ray structural analyses at 100 K to determine their molecular structures and arrangement patterns.

**Table 1:** Guest formula in **H*_n_* ImTP*^n^* ^+^** Cl^−^ *_n_* •(Guest) for as‐grown single‐crystals and powder sample stored under air after vacuum drying.

	As‐grown^[^ ^a^ ^]^	Powder under air ^[^ ^b^ ^]^
**ImTP**	1(Et_2_O), 3(H_2_O)	3.5(H_2_O)
**H_1_ImTP^+^**	2(CHCl_3_)	4.0(H_2_O)
**H_2_ImTP^2+^**	4(C_4_H_8_O)	4.0(H_2_O)
**H_4_ImTP^4+^**	—	6.0(H_2_O)

^[a]^ Formula determined by single‐crystal X‐ray crystal structural analysis at 100 K.

^[b]^ Formula determined by TG measurements of powder samples stored under atmospheric conditions after vacuum drying and elemental analysis.

The thermal stabilities of the as‐grown samples **ImTP**•(Et_2_O)•3(H_2_O), **H_1_ImTP⁺**•Cl^−^•2(CHCl_3_), and **H_2_ImTP^2^⁺**•Cl^−^_2_•4(THF) were low around 298 K due to rapid elimination of guest molecules (Et_2_O, CHCl_3_, and THF). The formula of the powder samples was determined from elemental analysis and TG measurements of the vacuum‐dried samples at 298 K and left under the air. Vacuum‐dried samples reabsorbed H_2_O under air, while the powder sample heated to 380 K and then cooled under N_2_ flow remained in an anhydrous state. TG charts of powder sample held under air after vacuum drying showed weight loss in all samples as the temperature increased from room temperature (Figure S3), confirming that the water molecules present in the powder samples were easily desorbed. Therefore, variable temperature PXRD measurements were performed using powder samples that were vacuum‐dried at 298 K under N_2_ flow, while the dielectric spectra were measured under the humidity‐ controlled condition.

Elemental analysis and TG measurements of the powder samples stored in air confirmed that they were **ImTP**•3.5(H_2_O), **H_1_ImTP** ^+^Cl^−^•4.0(H_2_O), **H_2_ImTP^2+^** Cl^−^_2_•4.0(H_2_O), and **H_4_ImTP^4+^** Cl^−^_4_•6.0(H_2_O).

Powder samples left at room temperature desorbed guest molecules present in the single‐crystal X‐ray structural analyses and reabsorbed H_2_O. In the TG measurement of **ImTP**•3.5(H_2_O) powder sample, a weight loss of 11.3% was observed at around 400 K, which was in good agreement with the theoretical value of 11.9% for 3.5 molecules of H_2_O. Similarly, **H_1_ImTP^+^** Cl^−^•4.0(H_2_O), **H_2_ImTP^2+^** Cl^−^_2_•4.0(H_2_O), and **H_4_ImTP^4+^** Cl^−^_4_•6.0(H_2_O) powder samples also showed weight losses of approximately 12.3, 11.6, and 15.2% around 400 K as the temperature increased, which agreed with the theoretical values of 12.6, 11.8, and 15.0% corresponding to the desorption of 4.0, 4.0, and 6.0 molecules of H_2_O, respectively. Further heating revealed weight loss associated with HCl desorption around 600 K, while **ImTP** remained stable within the temperature range up to 750 K. The differential scanning calorimetry (DSC) measurements of the powder sample stored in air showed a change in the baseline around 360−370 K, where water molecule desorption begins, and no clear phase transition peak was observed (Figure S4).

### 2.2 Crystal Structures of H_n_ImTP^n+^Cl^−^_n_

To investigate how the molecular structure and arrangement pattern of **ImTP** change with stepwise protonation, single crystal X‐ray structural analysis was used to evaluate the crystal structures for *n* = 0, 1, and 2 of **H*_n_* ImTP*^n^* ^+^Cl^−^ *_n_*** (Table S1). The space groups of **ImTP**•(Et_2_O)•3(H_2_O), **H_1_ImTP⁺**Cl^−^•2(CHCl_3_), and **H_2_ImTP^2^⁺**Cl^−^_2_•4(THF) were *C*2/*c*, *C*2/*c*, and *I*‐42*d*, respectively. The nonplanar molecules **ImTP**, **H_1_ImTP^+^**, and **H_2_ImTP^2+^** adopted a saddle‐shaped conformation in which the four benzimidazole units alternately oriented upward and downward. This conformation is consistent with that observed in typical **TP** molecules. The dihedral angles between adjacent benzimidazole units in **ImTP**, **H_1_ImTP^+^**, and **H_2_ImTP^2+^** were 59°, 70°, and 64°, respectively, with the degree of molecular distortion increasing in the order **H_1_ImTP^+^** > **H_2_ImTP^2+^** > **ImTP** (Figure S5).

**Figure 1:**
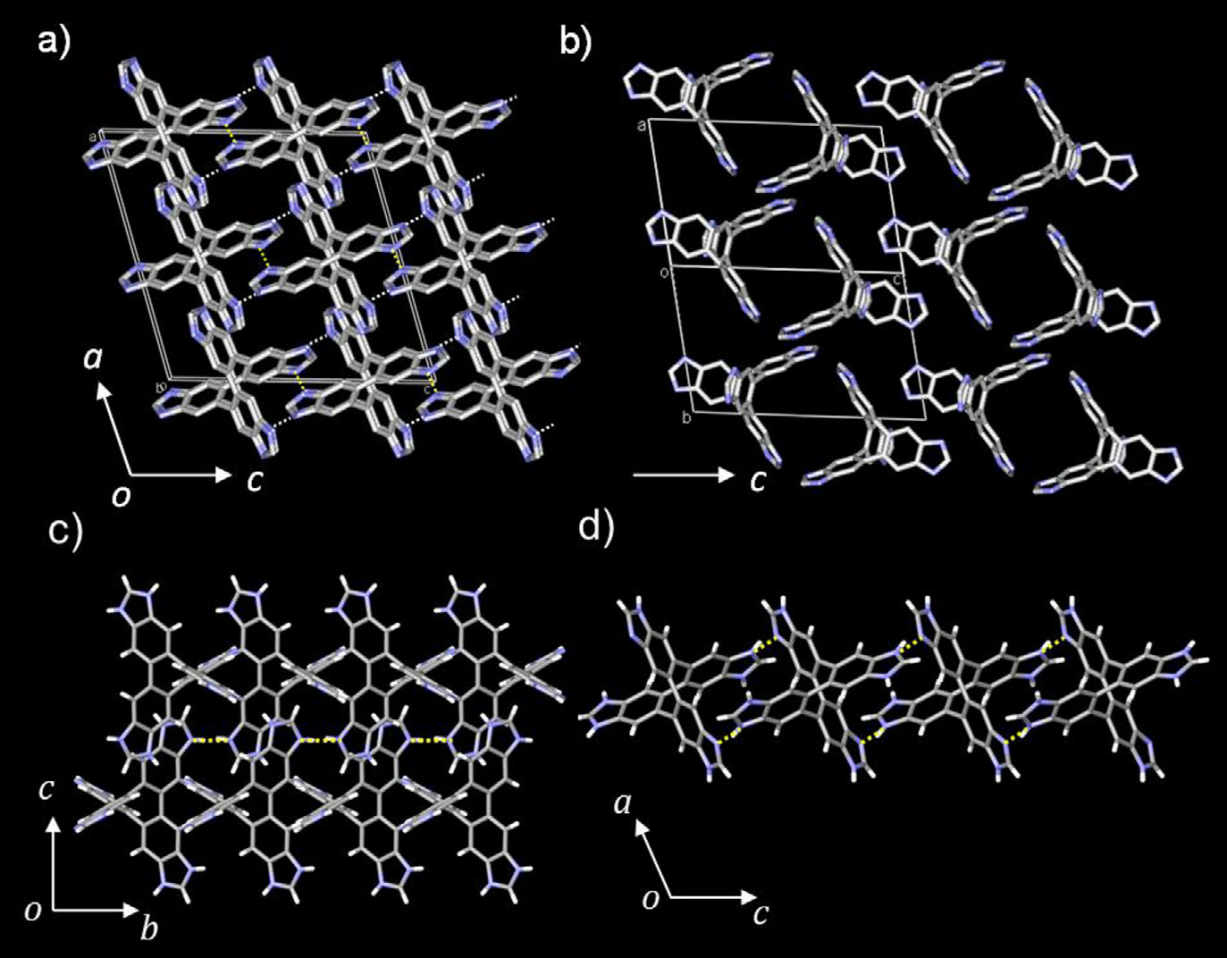
Crystal structures of **ImTP**. a) Unit cell viewed along the *b*‐axis and b) along the *a *+ *b* axis. Guest molecules of Et_2_O and H_2_O were omitted in figure. Hydrogen‐bonding viewed c) along the *a*‐axis and d) along the *b* axis.

**Figure 2:**
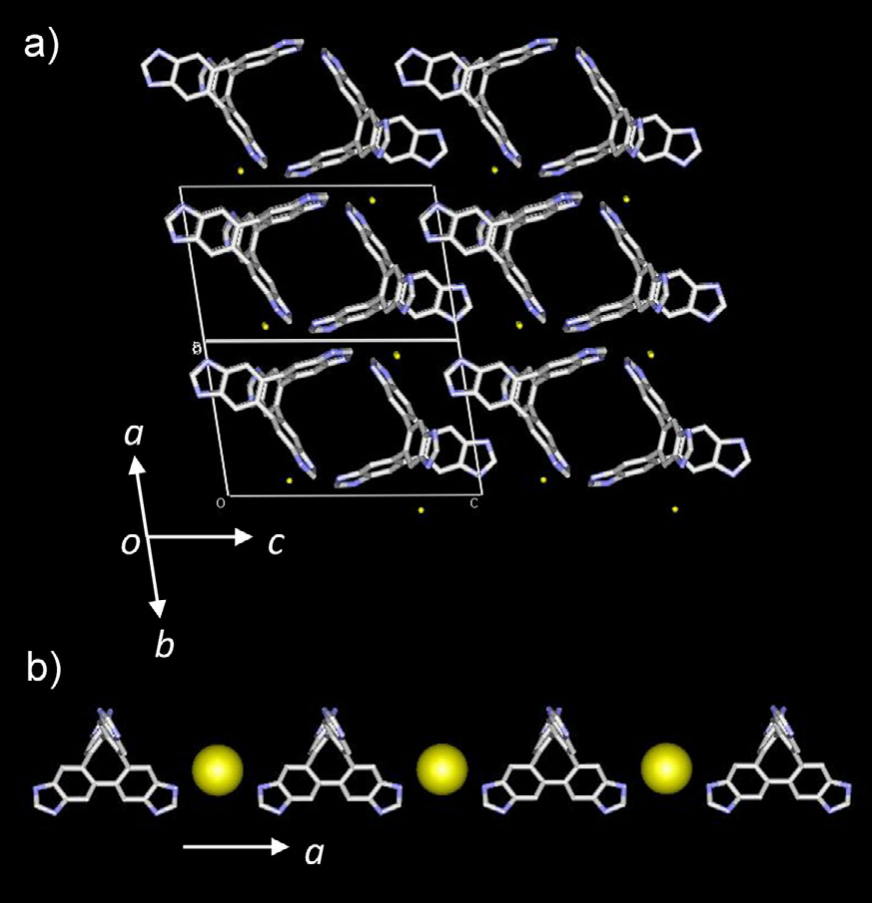
Crystal structures of **H_1_ImTP^+^Cl^−^** •2(CHCl_3_). a) Unit cell viewed along the *a *+ *b* axis. b) Electrostatic N─H•••Cl^−^ interaction along the *a*‐axis. Solvent molecules of CHCl_3_ were omitted in figure.

**Figure 3:**
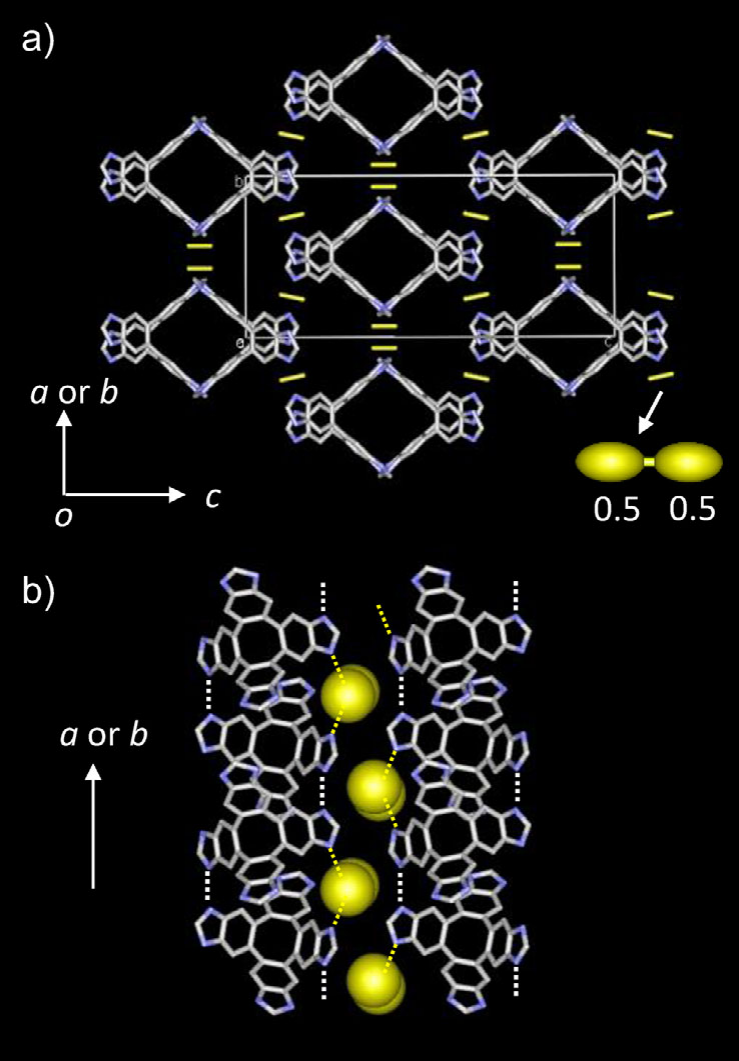
Crystal structures of **H_2_ImTP^+^Cl^−^_2_** •4(Et_2_O). a) Unit cell viewed along the *a*‐ or *b*‐axis. Disordered Cl^−^ anions exist at an equal occupancy factor of 0.5:0.5. b) Intermolecular N─H•••N hydrogen‐bonding and electrostatic N─H^+^•••Cl^−^ interactions along the *a‐* and *b*‐axis.

Figures 1, 2, and 3 summarize the crystal structures of **ImTP**•(Et_2_O)•3(H_2_O), **H_1_ImTP^+^** Cl^−^•2(CHCl_3_), and **H_2_ImTP^2+^** Cl^−^_2_•4(THF), respectively. In the **ImTP**•(Et_2_O)•3(H_2_O) crystal, half of the **ImTP** molecule was the crystallographically independent unit (Figure S6). Since there were no electrostatic interactions in the crystal, the crystal lattice was formed by N─H···N hydrogen bonding interactions between the benzimidazole moieties (Figure 1a). The intermolecular hydrogen bond N···N distances (*d*_N─N_) were 2.780 and 2.806 Å, which were comparable in strength to those observed in other **Im** groups (*d*_N─N_ = 2.835 Å).^[^ ^42^ ^]^ In contrast, benzimidazole tends to adopt a layered arrangement due to strong π–π interactions, exhibiting larger *d*_N–N_ values over 3.5 Å.^[^ ^43^ ^]^ In the case of **ImTP**, steric hindrance reduced π–π stacking interactions, thereby shortening the distance and enhancing the strength of N···N hydrogen bonding interactions. **ImTP** molecules are arranged along the *b*‐axis with molecular twisting, forming a 1D chain along the *b*‐axis via double intermolecular N─H···N hydrogen bonding chains (*d*_N─N _= 2.780 Å) (Figure 1c). The double hydrogen bonded chains interacted along the *c*‐axis via N─H···N hydrogen bonds (*d*_N─N_ = 2.806 Å) (Figure 1d), forming a cross‐lattice‐like arrangement, with one molecule of Et_2_O and three molecules of H_2_O introduced into the space. The void space occupied in the unit cell was 39%, and the space surrounded by the π electrons of benzimidazole formed a 1D channel along the *a *+ *b* axis (Figure S7).

The monoprotonated single‐crystal **H_1_ImTP^+^** Cl^−^•2(CHCl_3_) belongs to the crystal space group *C*2/*c* and has the same structure as the neutral **ImTP** single‐crystal. Half of the **H_1_ImTP^+^** cations are crystallographically independent (Figure S8), and two molecules of CHCl_3_ are contained as the crystallization solvent. Since one of the four benzimidazole units is protonated to form a benzimidazolium cation, the crystal lattice of **H_1_ImTP^+^** •Cl^−^ host exhibits both electrostatic interactions and hydrogen bonding. Along the *b*‐axis of the unit cell, the formation of 2D intermolecular N─H···N hydrogen bonds similar to **ImTP** was confirmed (Figure S9), with intermolecular hydrogen bonds formed at *d*_N─N_ = 2.743 and 2.725 Å. Figure 2a shows the unit cell viewed along the *a *+ *b* axis. The formation of a 1D channel similar to that of neutral **ImTP** is observed along the *a *+ *b* axis. A significant difference from the neutral **ImTP** single crystal is that electrostatic N─H^+^···Cl^−^ interactions are observed along the *a*‐axis of the unit cell with a N···Cl interatomic distance *d*_Cl─N_ = 3.179 Å (Figure 2b). The intermolecular interactions along the *a*‐axis were stronger through electrostatic interactions than through neutral N─H···N intermolecular hydrogen bonds, resulting in the formation of a more robust 2D network structure. The void space within the unit cell was estimated to be 30%, which is smaller than the 39% in the neutral **ImTP** single crystal (Figure S10). This is believed to be due to the formation of a more robust 2D network structure.

In the diprotonated **H_2_ImTP^2+^** Cl^−^_2_•4(THF) single‐crystal, the symmetry of the crystal increased and the space group changed to *I*‐42*d*. One‐quarter of the **H_2_ImTP^2+^** dication was crystallographically independent structural unit (Figure S11). The Cl^−^ anion had a site occupancy of 0.5:0.5 and was disordered. The basic molecular arrangement is similar to that of the crystal lattice formed by neutral **ImTP** or monocationic **H_1_ImTP^+^** molecules. Figure 3a shows the unit cells viewer along the *a‐* and *b*‐axis. Bent **H_2_ImTP^2+^** molecules formed a 1D channel structure facing each other, extending along the *a*‐ and *b*‐axes of the crystal (Figure S12). Between **H_2_ImTP^2^⁺** molecules, both N─H···N hydrogen bonds and N─H⁺···Cl^−^ electrostatic hydrogen bonds formed a 3D network structure.

Along the *a*‐ and *b*‐axes of the unit cell, intermolecular N─H···N hydrogen bonds were observed at *d*_N─N_ = 2.653 Å, which is shorter than the values observed in **ImTP** and **H_1_ImTP^+^** single crystals, indicating the formation of strong intermolecular hydrogen bonds. Furthermore, along the *a*‐ and *b*‐ axes of the unit cell, N─H^+^···Cl^−^ electrostatic hydrogen bonds with *d*_Cl–N_ = 2.982 and 3.158 Å were present, forming a strong 2D network structure between molecules. Intermolecular interactions mediated by N─H^+^···Cl^−^ interactions were also observed along the *c*‐axis, indicating that the crystal lattice of **H_2_ImTP^2+^** was formed through 3D intermolecular interactions. Since the channel extends in two directions of the crystal, the void space occupied by Et_2_O molecules accounted for 47% of the unit cell. This is a larger value compared to the void spaces present in **ImTP** and **H_1_ImTP^+^** crystals. The network of the **H*_n_* ImTP*^n^* ^+^** host lattice tended to increase in its dimensionality and become more robust through stepwise protonation. Since it was not possible to obtain a single crystal of **H_4_ImTP^4+^** Cl^−^_4_, discussions regarding the crystal lattice formed by the tetracation could not be conducted. However, the formation of a 3D network structure via N─H^+^···Cl^−^ interactions is predicted.

### 2.3 Stability of Hydrogen‐Bonding Crystal Lattice After Guest Desorption

Single‐crystals containing guest molecules are stable at low temperatures around 100 K, while guest molecules easily desorb at room temperature. Therefore, we evaluated the structural stability associated with guest molecule desorption from the hydrogen‐bonding host lattice formed by **H*_n_* ImTP*^n^* ^+^** and Cl^−^ using variable‐temperature PXRD measurements under N_2_. Neutral **ImTP** single‐crystals form a host lattice through 2D intermolecular N─H···N hydrogen bonds, with no contribution from electrostatic interactions. The PXRD patterns at 100 and 300 K are in good agreement with each other and also consistent with simulations derived from the single‐crystal X‐ray crystal structure at 100 K (Figure S13). Upon heating to 380 K, the PXRD pattern is nearly identical to that at 300 K, and the high‐angle Bragg diffraction peaks are slightly broadened. However, even after the guest molecules have desorbed, the host lattice formed by the hydrogen‐bonding **ImTP** molecules is considered to remain stable.

**Figure 4:**
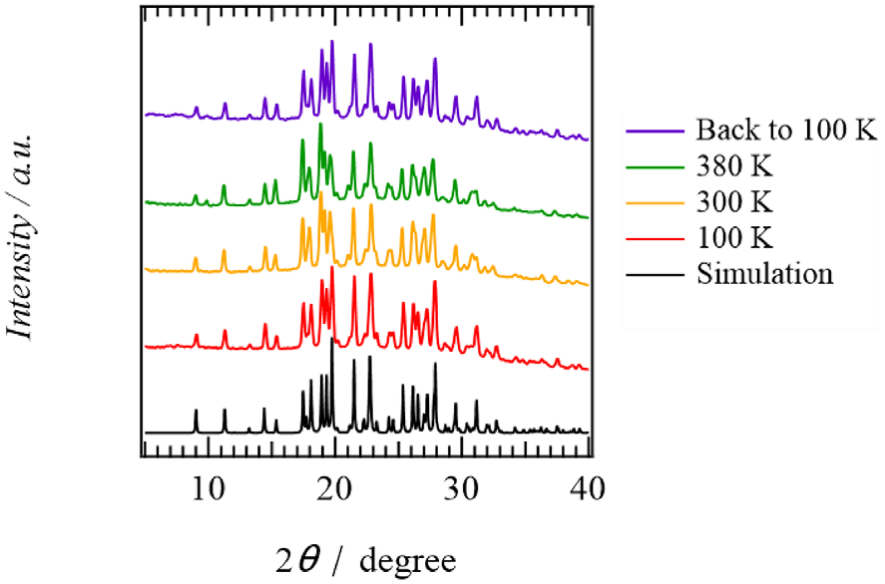
Variable‐temperature PXRD patterns under N_2_ flow of **H_1_ImTP^+^Cl^−^**. Simulation of **H_1_ImTP^+^Cl^−^** •2(CHCl_3_) single crystal (black) and PXRD patterns at *T* = 100 K (red), 300 (yellow), 380 K (green), and returned to 100 K (purple).

Figure 4 shows the variable PXRD patterns of **H_1_ImTP^+^** Cl^−^•2(CHCl_3_). The simulation pattern based on single‐crystal X‐ray crystallographic analysis at 100 K closely matches the PXRD pattern at 100 K, and no significant changes were observed at 300 K. Even at 380 K, where two CHCl_3_ molecules have completely desorbed, the PXRD pattern at 380 K closely matches the PXRD pattern at 100 K. Furthermore, the PXRD pattern of the sample cooled from 380 K back to 100 K after heating undfer N_2_ flow also showed no significant changes. Upon heating to 380 K, guest included **H_1_ImTP^+^** Cl^−^•2(CHCl_3_) is thought to have transformed into guest‐free **H_1_ImTP^+^** Cl^−^ due to desorption of readsorbed H_2_O, where the CHCl_3_ or H_2_O molecules acting as guests have desorbed. However, the 2D N─H···N hydrogen bonds and N─H^+^···Cl^−^ electrostatic interactions maintained a stable network structure, preserving the high crystallinity of the host lattice.

Similarly, the variable‐temperature PXRD patterns of the diprotonated **H_2_ImTP^2+^** Cl^−^_2_ showed a diffraction pattern that differed from the simulated pattern obtained from single‐crystal X‐ray structural analysis of **H_2_ImTP^2+^** Cl^−^_2_•4(Et_2_O) at 100 K. The PXRD patterns at 100 and 300 K were consistent with each other, while they did not match the simulated PXRD pattern derived from the single‐crystal X‐ray crystal structural analysis at 100 K (Figure S14), indicating that the host lattice had changed due to the desorption of the guest Et_2_O molecule. When heated to 380 K under N_2_ flow, the diffraction pattern changed from the 300 K pattern, and no significant changes were observed even when cooled back to 100 K. Sharp Bragg reflection peaks were observed at all measurement temperatures, suggesting that high crystallinity was maintained. While the desorption of guest molecules from the as‐grown crystal maintains crystallinity, the arrangement pattern of **H_2_ImTP^2+^** Cl^−^_2_ is believed to have changed.

Furthermore, the variable‐temperature PXRD patterns of protonated **H_4_ImTP^4+^** Cl^−^_4_ show only broad Bragg reflections around 2*θ* = 5∼40° without sharp diffraction peaks at 100, 300, and 380 K (Figure S15). Therefore, the solid state **H_4_ImTP^4+^** Cl^−^_4_, where electrostatic interactions dominate, is in an amorphous state with low crystallinity, consistent with the fact that it was difficult to prepare single crystals. By gradually protonating **ImTP**, increasing the contribution of electrostatic interactions of N─H^+^···N and N─H^+^···Cl^−^ to intermolecular neutral N─H···N hydrogen bonds, very high crystallinity independent of guest molecule adsorption/desorption processes was confirmed at protonation number of *n* = 1. The increase in intermolecular interactions due to electrostatic interactions does not necessarily contribute to the stabilization of the host lattice by the adsorption and desorption of guest molecules, and the balance between N─H···N hydrogen bonds and electrostatic interactions is important. The increase in the contribution of electrostatic interactions stiffens the host lattice and reduces its flexibility to structural changes in the host lattice associated with the adsorption‐desorption of guest molecules. The water adsorption site is presumed to be the hydrogen‐bonding network of the **Im** moiety, which exhibits high affinity for water. When guest molecules desorb from the single crystal and H_2_O readsorbs, no significant changes in the host lattice are observed in the PXRD patterns, except for **H_4_ImTP^4+^** Cl^−^_4_. Therefore, it is considered that the original guest molecule sites in the single crystal sample become the readsorption sites for H_2_O.

### 2.4 H_2_O Adsorption/Desorption Behavior

PXRD measurements showed that the crystallinity and reversibility of molecular assembly structures vary depending on the number of protons introduced in **H*_n_* ImTP*^n^* ^+^** Cl^−^ *_n_*. Among these (*n* = 0, 1, 2, and 4), the crystal formed by **H_1_ImTP^+^** with *n* = 1 showed the highest stability. Furthermore, no highly crystalline samples were obtained for *n* = 4, and they were amorphous.

**H*_n_* ImTP*^n^* ^+^** Cl^−^ *_n_* •*x*(H_2_O) was pretreated at 398 K under vacuum to completely remove H_2_O molecules, and the adsorption isotherms of N_2_ at 77 K and H_2_O at 298 K of anhydrous **H*_n_* ImTP*^n^* ^+^** •Cl^−^ *_n_* were evaluated. For N_2_, no adsorption behavior was observed at *n* = 0 and 4 (Figure S16). On the other hand, for *n* = 1 and 2, adsorption amounts of approximately *n*_a_ = 2.5 and 1.0 mol mol^−1^ were confirmed at a relative pressure *P*/*P*_0_ = 1, with BET surface areas of 60.6 and 10.3 m^2^ g^−1^, respectively. This result is consistent with the fact that the structural flexibility and reversibility of the host lattice, as determined from PXRD, are higher for *n* = 1 and 2 and lower for *n* = 0 and 4.

**Figure 5:**
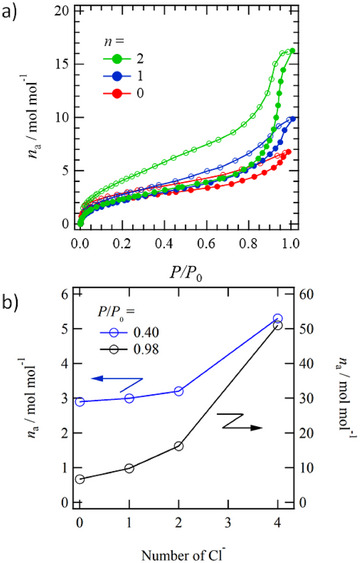
H_2_O sorption isotherm of anhydrous **H*_n_* ImTP*^n^* ^+^** Cl^−^ *_n_* at 298 K (*n* = 0, 1, and 2). a) The *n*‐dependent *n*_a_ versus *P*/*P*_0_ plots (*n* = 0, 1, and 2). b) The *n*‐dependent *n*_a_ versus number of Cl^−^ at *P*/*P*_0_ = 0.40 and 0.98.

The results of elemental analyses and TG measurements of **H*_n_* ImTP*^n^* ^+^** Cl^−^ *_n_*, which were vacuum dried at room temperature and kept under atmospheric conditions, showed readsorption of H_2_O molecules, with H_2_O amounts (*x*) of 4.0, 3.3, 3.5, and 5.5 for *n* = 0, 1, 2, and 4, respectively. Figure 5 shows the H_2_O adsorption isotherms of activated **H*_n_* ImTP*^n^* ^+^** Cl^−^ *_n_* at 298 K, excluding *n* = 4 (Figure S17). From the PXRD patterns, it was confirmed that *n* = 0 and 1 retain the hydrogen‐bonding network structure observed in the single‐crystals, while *n* = 2 exhibits a slight structural change while maintaining high crystallinity. For **H*_n_* ImTP*^n^* ^+^** Cl^−^ *_n_* with *n* = 0, 1, and 2, the H_2_O adsorption amount (*n*_a_, mol mol^−1^) indicated by *n*_a_ increases immediately with an increase in *P*/*P*_0_, reaching ∼3 around *P*/*P*_0_ = 0.4, which agrees with the H_2_O amount determined from elemental analysis. Additionally, at *n* = 4, the *n*_a_ value increased to approximately ∼5 near *P*/*P*_0_ = 0.4, and this value also matched the results of elemental analysis. Therefore, it is considered that after the guest molecules contained in the as‐grown single crystals, such as Et_2_O or CHCl_3_, have desorbed, H_2_O molecules are taken up from the atmosphere, and a stable composition capable of maintaining the host lattice structure exists. H_2_O adsorption in the *P*/*P*_0_ < 0.4 region is considered to be adsorption of H_2_O into 1D channels within the crystal.

Increasing *P*/*P*_0_ to 0.4 or higher causes *n*_a_ to increase gradually, reaching *n*_a_ = 5, 10, and 16 at *P*/*P*_0_ = 0.98 for *n* = 0, 1, and 2, respectively. This is thought to correspond to the adsorption and condensation of H_2_O molecules onto the crystal surface. The observation of hysteresis in both the adsorption and desorption processes suggests that H_2_O adsorption occurs in the 1D channel. Additionally, for **H_4_ImTP^4+^** Cl^−^_4_ with *n* = 4, the increase in *n*_a_ reaches nearly 50 as *P*/*P*_0_ = 0.98 (Figure S17), suggesting that surface H_2_O condensation occurs in the amorphous state. Therefore, it is considered that stable H_2_O molecule adsorption and desorption occur while maintaining the previously described hydrogen‐bonded crystal structure up to approximately *P*/*P*_0_ = 0.6.

### 2.5 The *n*‐Dependent Dielectric Response and Proton Conductivity

The frequency and temperature dependence of the dielectric constant varies greatly depending on the protonation and hydration state of the **HnImTP*^n^* ^+^** Cl^−^ *_n_* •*x*(H_2_O) (Figures S18 − 31). When pellet samples are exposed to air, they adsorb H_2_O. Therefore, dielectric measurements were performed under controlled humidity conditions. The amount of H_2_O adsorbed at the set humidity was estimated from the adsorption isotherm of H_2_O at 298 K. Table 2 summarizes the hydration number *x* estimated from the adsorption isotherms of the **H*_n_* ImTP*^n^* ^+^** Cl^−^ *_n_* •*x*(H_2_O) at the relative humidities RH = 0%, 40%, and 98%, as well as proton conductivity (σ_H+_, S cm^−1^), and activation energy (*E*_a_, eV). The dielectric properties at RH = 98% are included as reference data, as they are considered to include contributions from H_2_O aggregated on the crystal surface.

**Table 2:** Proton conducting properties of **H*_n_* TPIm*^n^* ^+^** Cl^−^ *_n_* •*x*(H_2_O) under RH = 0%, 40%, and 98%.

	RH	*x* ^[^ ^a^ ^]^	σ_H+_, S cm^−1^ (*T*, K)^[^ ^b^ ^]^	*E*_a_, eV
**ImTP**	0	0	−	−
	40	2.9	3.20 × 10^−^^7^ (363 K)	0.49(1)
	98	6.7	1.09 × 10^−^^4^ (363 K)	0.66(6)
**H_1_TPIm** ^1+^	0	0	−	−
	40	3.0	3.38 × 10^−^^6^ (363 K)	0.50(1)
	98	9.8	1.64 × 10^−^^3^ (363 K)	0.30(1)
**H_2_TPIm** ^2+^	0	0	4.12 × 10^−^^8^ (370 K)	0.79(2)
	40	3.2	1.35 × 10^−^^6^ (363 K)	0.74(1)
	98	16	3.71 × 10^−^^3^ (363 K)	0.38(0)
**H_4_TPIm** ^4+^	0	0	4.19 × 10^−^^7^ (370 K)	0.81(2)
	40	5.3	6.20 × 10^−^^6^ (363 K)	0.87(2)
	98	51	2.00 × 10^−^^3^ (363 K)	0.39(1)

^[a]^ Determined by H_2_O isotherm at 298 K. The initial measurement of the vacuum‐dried sample represents the result under conditions of RH = 0%.

^[b]^ Measured by a humidity‐ controlled condition.

**Figure 6:**
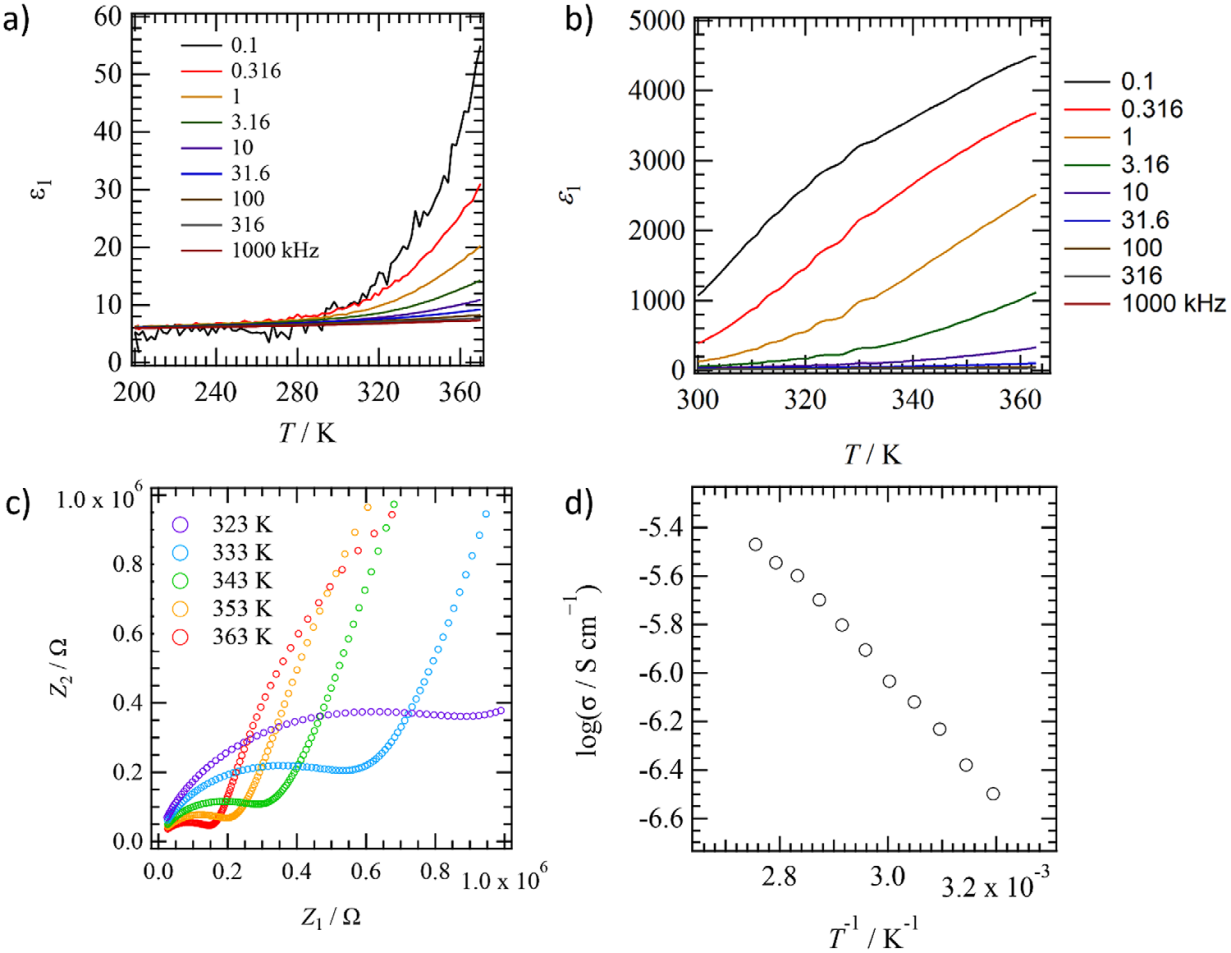
Dielectric responses and proton conductivity of **H*_2_* ImTP*^2^* ^+^** Cl^−^_2_. *T*‐ and *f*‐dependent *ε*_1_ a) under RH = 0% and b) under RH = 40%. c) *T*‐dependent Nyquist plot and d) Arrhenius plot of σ_H+_ of **H_2_ImTP^2+^** Cl^−^_2_ under RH = 40%.

Figure 6a,b show the temperature (*T*) and frequency (*f*) dependence of the real part of the dielectric constant (*ε*_1_) under conditions of RH = 0% (anhydrous state) for **H_2_ImTP^2+^** Cl^−^_2_ and RH = 40% for **H_2_ImTP^2+^** Cl^−^_2_, respectively. Anhydrous **H_2_ImTP^2+^** Cl^−^_2_ under RH = 0% showed constant values for both *ε*_1_ and *ε*_2_ over a *T* sweep from 200 to 360 K and did not exhibit *T*‐ and *f*‐dependence (Figure S18). On the other hand, for **H_2_ImTP^2+^** •Cl^−^_2_•4.0(H_2_O) at RH = 40%, *ε*_1_ at 300 K and *f* = 100 Hz showed a large value of approximately 1000, with a significant increase in *ε*_1_ at low *f* values, reaching approximately *ε*_1_ = 4500 at 360 K. The value of imaginary part of the dielectric constant *ε*_2_ also showed a large value, confirming the presence of dipole moment dynamics in the molecular aggregate (Figure S25).

Figure 6c shows the temperature dependence of the Nyquist plot using resistance (*Z*_1_) and susceptance (*Z*_2_) in **H_2_ImTP^2+^** Cl^−^_2_•4.0(H_2_O) under RH = 40%. A semicircular trace in the Nyquist plots was observed, which is thought to be due to the contribution of σ_H+_, and the semicircle became smaller as the temperature increased. At 363 K, the σ_H+_ value reached 1.35 × 10^−6^ S cm^−1^. The log σ_H+_ – *T* ^−1^ plot created from the *T*‐dependence of σ_H+_ exhibits Arrhenius behavior (Figure 6d), with an activation energy *E*_a_ of 0.74(1) eV. At RH = 0%, a semicircular trace in the Nyquist plots was observed in anhydrous **H_2_ImTP^2+^** Cl^−^_2_, and σ_H+_ = 4.12 × 10^−^^8^ S cm^−1^ at 370 K with *E*_a_ of 0.79(2) eV. At RH = 98%, σ_H+_ at 363 K increased to 3.71 × 10^−3^ S cm^−1^, which includes the contribution of conductivity due to H_2_O aggregated on the compressed pellet surface, and the activation energy also decreased to *E*_a_ = 0.38(0) eV (Figure S38). It is considered that the σ_H+_ increased by a factor of 100 due to the formation of a proton conduction path when H_2_O was incorporated into the channel as a guest molecule in the ionic host lattice formed by **H_2_ImTP^2^⁺**.

The proton conducting switching was observed **H_2_ImTP^2+^** Cl^−^_2_ under RH = 40%, where σ_H⁺_ of 3.38 × 10^−^^6^ S cm^−1^ was observed at 363 K and the *E*_a_ of 0.50(1) eV. In neutral **ImTP**, no dielectric response was observed under anhydrous conditions at RH = 0%, and no σ_H+_ was detected (Figure S18). Under humidified conditions at RH = 40%, the σ_H+_ value at 363 K was 3.20 × 10^−7^ S cm^−1^ and *E*_a_ was calculated to be 0.49(1) eV (Figure S32). At RH = 98%, approximately seven molecules of H_2_O were adsorbed, and at 363 K, σ_H+_ was 1.09 × 10^−4^ S cm^−1^, with *E*_a_ = 0.66(6) eV (Figure S33). Since the amount of H_2_O adsorption is smaller compared to *n* = 1, 2, and 4, it is considered that the amount of H_2_O adsorbed on the surface is also smaller. It is speculated that the conductivity of σ_H+_ = 10^−4^ S cm^−1^ is manifested due to the contribution of H_2_O introduced into the channel to proton conductivity.

In anhydrous **H_1_ImTP^+^** Cl^−^ under RH = 0%, no semicircular trace was observed in the Nyquist plot, and no σ_H+_ was exhibited. Under RH = 40% conditions, the composition became **H_1_ImTP^+^** Cl^−^•3(H_2_O) with three H_2_O molecules adsorbed, and the σ_H+_ value was confirmed. The σ_H+_ at 363 K was 3.38 × 10^−6^ S cm^−1^ (Figure S34), which was one order of magnitude higher than that of neutral **ImTP** under the same conditions. On the other hand, the activation energy was 0.50 eV, which was the same as that of neutral **ImTP** at RH = 40%. Since **ImTP** and **H_1_ImTP^+^** Cl^−^•3(H_2_O) have nearly identical 1D channel‐type crystal structures, the tenfold increase in σ_H+_ value is attributed to the introduction of H^+^ as carriers. Under RH = 98% conditions, a high proton conductivity of σ_H+_ = 1.64 × 10^−3^ S cm^−1^ at 363 K and a low activation energy of *E*_a_ = 0.30 eV were observed (Figure S35), which is estimated to be due to the contribution of water molecules aggregated on the surface of the compressed pellet for the proton conductivity measurements.

The σ_H+_ value of fully protonated **H_4_ImTP^4+^** Cl^−^_4_ under anhydrous conditions of RH = 0% was σ_H+_ = 4.19 × 10^−7^ S cm^−1^ at 400 K, and the activation energy was *E*_a_ = 0.81(2) eV (Figure S38). The *E*_a_ value was nearly identical to that of **H_2_ImTP^2+^** Cl^−^_2_ under RH = 0% conditions (Figure S36), while the σ_H+_ improved by an order of magnitude, which is attributed to an increase in the number of H^+^ carriers. Under RH = 40% conditions, the σ_H+_ value of **H_4_ImTP^4+^** Cl^−^_4_ was σ_H+_ = 6.20 × 10^−6^ S cm^−1^ at 363 K, with an activation energy of *E*_a_ = 0.87 eV (Figure S40). This value is nearly equivalent to that measured for **H_2_ImTP^2+^** Cl^−^_2_ under the same conditions (Figurer S37). At RH = 98%, H_2_O aggregation on the surface of **H_4_ImTP^4+^** Cl^−^_4_ resulted in a high proton conductivity of σ_H+_ = 2.00 × 10^−3^ S cm^−1^, which is not an intrinsic property (Figure S41).

**Figure 7:**
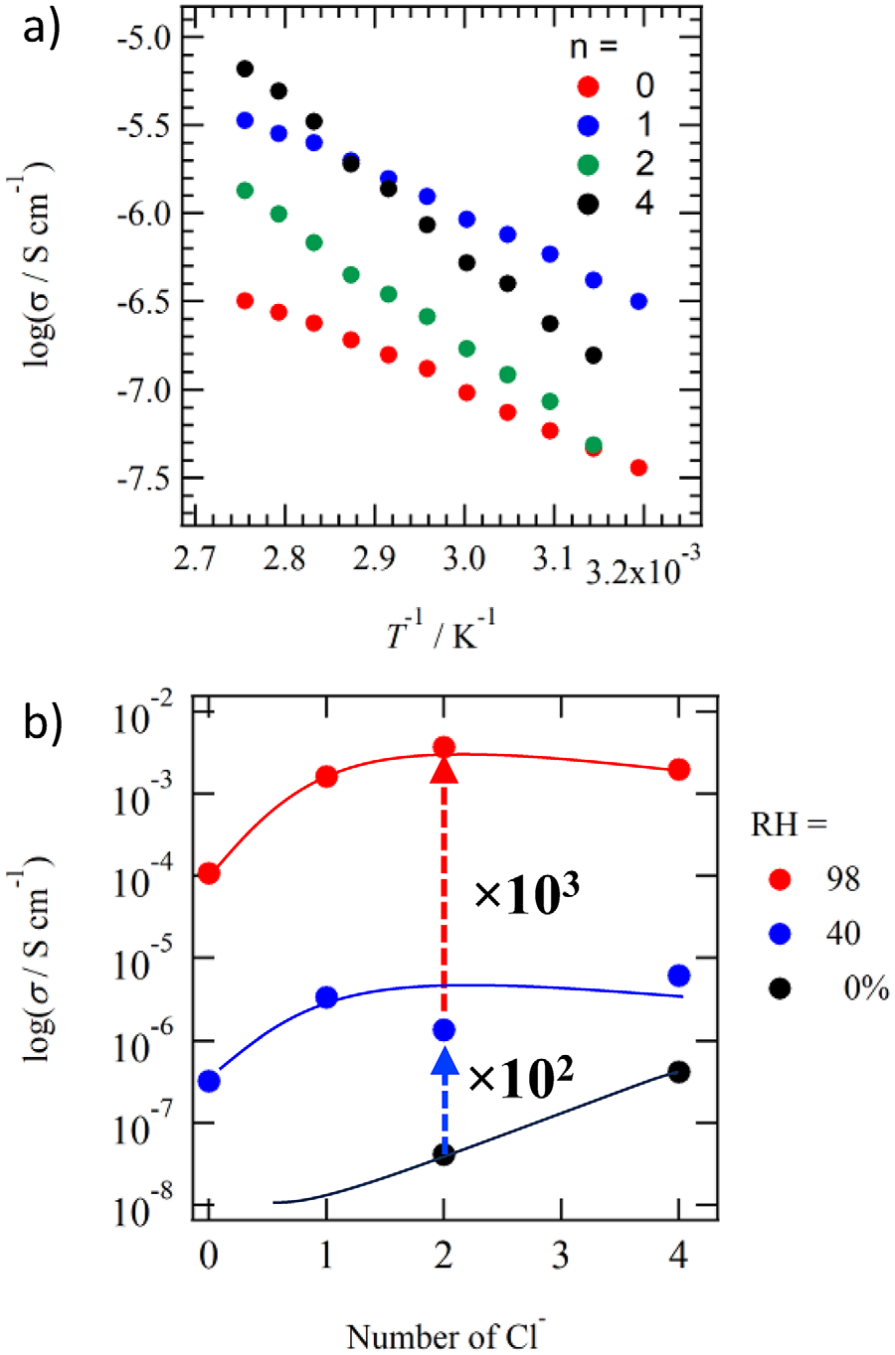
*T*‐ and *n*‐dependent proton conducting behaviors. a) The *n*‐dependent log σ_H+_ – *T* ^−1^ plots of **H*_n_* ImTP*^n^* ^+^** Cl^−^ *_n_* •*x*(H_2_O) under RH = 40%. b) The *n*‐dependent σ_H+_ values of **H*_n_* ImTP*^n^* ^+^** Cl^−^ *_n_* •*x*(H_2_O) under RH = 0%, 40%, and 98%. The solid lines are guides to the eyes.

Figure 7 shows the change in σ_H+_ values associated with stepwise protonation. Under conditions of RH = 40%, it is considered that H_2_O is incorporated as a guest molecule into the crystal lattice formed by **H*_n_* ImTP*^n^* ^+^** Cl^−^ *_n_* through hydrogen‐bonding and electrostatic interactions. Under such conditions, the σ_H+_ exhibited values ranging from 10^−7^ to 10^−6^ S cm^−1^ in the temperature range of 360–370 K. As a general trend, it was found that increasing the number of protons *n* acting as H^+^ carriers in neutral **ImTP** enhances the σ_H+_. Regarding the magnitude of *E*_a_ value, it increases in the order *n* = 4 > 2 > 0 ≈ 1, which depends on the maintenance of crystallinity and its reversibility during the adsorption and desorption of H_2_O molecules in the host lattice. As the protonation degree *n* increased, crystallinity decreased, and amorphization was confirmed particularly at *n* = 4. When crystallinity of the host structure decreased in response to water adsorption/desorption, a tendency toward increased activation energy was observed.

## 3 Conclusion

By introducing four **Im** groups into the **TP** π‐core of the **ImTP** molecule and performing stepwise protonation (*n* = 0, 1, 2, and 4) for **H*_n_* ImTP*^n^* ^+^**, we found that the balance between hydrogen‐bonding and electrostatic interactions can be precisely controlled, resulting in changes in the dimensionality and stability of the crystal structure. In the monoprotonated form **H_1_ImTP^+^**, we clarified that the optimal balance between hydrogen‐bonding and electrostatic interactions enables both high structural stability and excellent proton conductivity of 3.38 × 10^−^^6^ S cm^−^^1^ at 363 K. In neutral **ImTP**, a 2D N─H···N hydrogen bond network dominates, whereas as protonation progresses, N─H^+^···Cl^−^ electrostatic interactions are introduced, leading to the formation of a 3D network structure in **H_2_ImTP^2+^** and amorphization in **H_4_ImTP^4+^**, demonstrating systematic structural changes. By elucidating the resulting changes in proton conductivity (*n* = 0 < 1 ≈ 2 < 4), this study successfully provided a new material design guideline for physical property control using the protonation degree parameter. This insight is applicable not only to proton conductivity but also to ion conductivity, dielectric properties, molecular recognition, and other functional materials, providing important design guidelines for the next‐generation energy devices and environmentally responsive smart materials.

## 4 Experimental Section

### Physical measurement

Elemental analyses were performed on a Microcoder JM10 at the Elementary Analysis Laboratory, Institute of Multidisciplinary Research for Advanced Materials, Tohoku University. NMR spectra were recorded on a Bruker Avance III 400 or a JEOL ECZ‐400S spectrometer. UV‐vis‐NIR and IR spectra were recorded on PerkinElmer Lambda 750 and Thermo Fisher Scientific Nicolet 6700 FT‐IR spectrophotometers, respectively. Thermogravimetric (TG) differential thermal analysis and DSC were conducted using a Rigaku Thermo plus TG8120 thermal analysis station and Mettler DSC1‐T and a heating and cooling rate of 5 K min^−1^ under nitrogen. *T*‐dependent dielectric constants under vacuum conditions were measured using the two‐probe AC impedance method from 100 Hz to 1 MHz (Hewlett–Packard, HP4194A) and the temperature controller of a Linkam LTS‐E350 system. The AC impedance measurements under humidified conditions were conducted in the frequency range of 100 Hz to 1 MHz (Wayne Kerr Electronics, 6440B), and temperature and humidity were controlled using a compact constant‐temperature and ‐humidity chamber (IW223, Yamato Scientific Co., Ltd.). Powder samples were pressed into pellets (3 mm in diameter), then coated with Ag paste on both sides and connected using gold wires (25 µm in diameter). The adsorption isotherms of H_2_O vapor were measured at 298 K using a BELSORP‐max II apparatus (MicrotracBEL), and nitrogen adsorption isotherms were measured at 77 K using the same instrument. Each sample was subjected to vacuum drying for pretreatment.

### Synthesis and thermal stability of ImTP (Scheme S1)

A mixture of **OATP** hydrochloride (101.2 mg, 141.3 µmol), triethyl orthoformate (167.6 mg, 1.13 mmol), and ZrCl_4_ (13.2 mg 56.6 µmol) in dry MeOH (4 mL) was stirred at room temperature for 4 hours. After completion of the reaction, MeOH was removed under reduced pressure. The residue was washed three times with EtOH. The resulting wet cake was dissolved in 20 mL water and NaHCO_3_ (190.0 mg, 2.26 mmol) was added to the solution. The solution immediately turned cloudy, and solid precipitate appeared. The precipitate was filtered and washed with water three times. The solid was dried under vacuum to give 58 mg (yield 88%). **ImTP** (2 mg) was dissolved in EtOH (3 mL) and diluted with diethyl ether (Et_2_O) by vapor diffusion to give yellow single crystals of **ImTP**•(Et_2_O)•3(H_2_O) in 2 − 3 days. Single crystals containing Et_2_O were unstable at room temperature, and solvent molecules easily removed, causing changes in formula of the crystal. Elemental analysis was performed using crystalline samples after drying under vaccum at 298 K, followed by storing in air at 298 K. Elemental analysis for **ImTP**•4(H_2_O). Calcd for C_28_H_16_N_8_•4(H_2_O): C, 62.68; H, 4.51; N, 20.88. Found C, 62.52; H, 4.51; N, 20.82. _1_H NMR (400 MHz, DMSO‐d6) *δ* 12.37 (s, 4H), 8.16 (s, 4H), 7.25–7.36 (br d, 8H).

### Preparation of H_n_ImTP^n+^Cl^−^_n_

In a Schlenk flask (50 mL) **ImTP** (50.0 mg, 107.6 µmol) was dissolved in EtOH (10 mL). Hydrochloric acid (35%–37%, 109.3 µL × *n*, 113.0 µmol × *n*) was added according to the value of *n* (the number of proton). Then the mixture was stirred at room temperature for 1 hour. After solvent was removed under reduce pressure, the solid was washed with water (for **H_1_ImTP** ^+^Cl^−^ and **H_2_ImTP^2+^** Cl^−^_2_) or EtOH (for **H_4_ImTP^4+^** Cl^−^_4_). Single crystals of **H_*n*_ImTP^*n*+^** Cl^−^_*n*_ were prepared as follows. **H_1_ImTP^+^** Cl^−^ (2 mg) was dissolved in EtOH (3 mL) and diluted by CHCl_3_ by vapor diffusion to give brown single‐crystals of **H_1_ImTP^+^** Cl^−^•2(CHCl_3_) in 5–7 days. **H_2_ImTP^2+^** Cl^−^_2_ (2 mg) was dissolved in EtOH (3 mL) and diluted with tetrahydrofuran (THF) by vapor diffusion to give brown single‐crystals of **H_2_ImTP^2+^** Cl^−^_2_•4(THF) in 2 weeks. Obtained single crystals were unstable at room temperature, and solvent molecules easily removed, causing changes in formula of the crystal. Elemental analysis was performed using crystalline samples after drying under vacuum at 298 K, followed by storing in air at 298 K. Elemental analysis of **H_1_ImTP^+^** Cl^−^. Calcd for C_28_H_17_N_8_Cl•3.3(H_2_O): C, 60.01; H, 4.24; N, 20.00. Found C, 60.16; H, 4.12; N, 19.72. ^1^H NMR (400 MHz, DMSO‐d_6_) *δ* 8.58 (s, 4H), 7.43 (s, 8H). **H_2_ImTP^2+^** Cl^−^_2_. Calcd for C_28_H_18_N_8_Cl_2_•3.5(H_2_O): C, 52.80; H, 4.11; N, 17.59. Found C, 53.02; H, 3.98; N, 17.32. ^1^H NMR (400 MHz, DMSO‐d_6_) *δ* 8.84 (s, 4H), 7.51 (s, 8H). It was not possible to obtain single crystals of **H_4_ImTP^4+^** Cl^−^_4_. Elemental analysis was performed using powder samples vacuum‐dried at 298 K. **H_4_ImTP^4+^** Cl^−^_4_. Calcd for C_28_H_20_N_8_Cl_4_•5.5(H_2_O): C, 47.41; H, 4.40; N, 15.80. Found C, 47.75; H, 4.08; N, 15.46. ^1^H NMR (400 MHz, DMSO‐d6) *δ* 9.20 (s, 4H), 7.62 (s, 8H).

### Crystal structural determination

Crystallographic data of **ImTP**•(Et_2_O)•3(H_2_O), **H_1_ImTP^+^** Cl^−^•2(CHCl_3_), and **H_2_ImTP^2+^** Cl^−^_2_•4(THF) were collected using a Rigaku RAPID‐II diffractometer equipped with a rotating anode fitted with a multilayer confocal optic and using Cu Kα (*λ*  = 1.54 187 Å) radiation from a graphite monochromator (Table S1). Structural refinements were performed using the full‐matrix least‐squares method on *F* ^2^. All the parameters, except for those of the hydrogen atoms, were refined using anisotropic temperature factors.

## Supporting Information

Experimental Section, ^1^H NMR spectra, TG charts, DSC charts, crystal structures, *T*‐dependent PXRD, N_2_ and H_2_O sorption isotherms, *T*‐ and *f*‐dependent dielectric constants, and proton conductivities.

## Conflict of Interest

The authors declare no conflict of interest.

## Supporting information



Supporting Information

Supporting Information

## Data Availability

Deposition numbers 2 472 294 (for **ImTP**•(Et_2_O)•3(H_2_O)), 2 472 295 (for **H_1_ImTP** ^+^Cl^−^•2(CHCl_3_)), and 2 472 296 (for H_2_ImTP^2+^Cl^−^_2_•4(THF)) contain the supplementary crystallographic data for this paper. These data are provided free of charge by the joint Cambridge Crystallographic Data Centre and Fachinformationszentrum Karlsruhe Access Structures service.
