# Supplementary material for: Proton Conductivities of Stepwise Protonated Imidazole‐Fused Tetraphenylene Derivatives
Source: Chemistry. 2025 Oct 9;31(62):e02622. doi: 10.1002/chem.202502622 (PMC12598376; doi:10.1002/chem.202502622)
Supplement: Supplementary file 1 — Supporting Information [file CHEM-31-e02622-s002.pdf]

Supporting Information

Proton Conductivities of Stepwise Protonated Imidazole-fused Tetraphenylene Derivatives

Mu Li,[a] Takashi Takeda,[a, b, c]* Shun Dekura,[a, b] Tetsu Sato,[a, b] and Tomoyuki Akutagawa [a, b]*

[a] M. Li, Prof. T. Takeda, Dr. S. Dekura, Dr. T. Sato, Prof. T. Akutagawa
Graduate School of Engineering
Tohoku University
Sendai 980-8579, Japan
E-mail: takashi@shinshu-u.ac.jp and akutagawa@tohoku.ac.jp

[b] Prof. T. Takeda, Dr. S. Dekura, Dr. T. Sato, Prof. T. Akutagawa
Institute of Multidisciplinary Research for Advanced Materials (IMRAM)
Tohoku University
2-1-1 Katahira, Aoba-ku, Sendai 980-8577, Japan

[c] Prof. T. Takeda
Faculty of Science
Shinshu University
3-1-1 Asahi, Matsumoto 390-8621, Japan

Contents

1. Experimental section.

2. n-dependent ^{1}H NMR spectra (Figures S1, 2).

3. TG charts (Figure S3).

4. DSC charts (Figure S4).

5. Non-planar **ImTP** molecule (Figure S5).

6. Crystal structure of **ImTP** (Figures S6, 7).

7. Crystal structure of **H_1ImTP$^+$**•Cl^-•2($CHCl_3$) (Figures S8, 9, 10).

8. Crystal structure of **H_2ImTP^{2+}**•Cl^-_2•4(THF) (Figures S11, 12).

9. *T*-dependent PXRD patterns (Figures S13, 14, 15).

10. N_2 sorption isotherm (Figure S16).

11. H_2O sorption isotherm (Figure S17)

12. *T*- and *f*-dependent dielectric constants (Figures S18-31).

13. *T*-dependent proton conductivity (Figures S32-41).

Experimental Section.

Physical measurements. Elemental analyses were performed on a Microcoder JM10 at the Elementary Analysis Laboratory, Institute of Multidisciplinary Research for Advanced Materials, Tohoku University. NMR spectra were recorded on a Bruker Avance III 400 or a JEOL ECZ-400S spectrometer. UV-vis-NIR and IR spectra were recorded on PerkinElmer Lambda 750 and Thermo Fisher Scientific Nicolet 6700 FT-IR spectrophotometers, respectively. Thermogravimetric (TG) differential thermal analysis and differential scanning calorimetry (DSC) were conducted using a Rigaku Thermo plus TG8120 thermal analysis station and Mettler DSC1-T and a heating and cooling rate of 5 K min^{-1} under nitrogen. *T*-dependent dielectric constants under vacuum conditions were measured using the two-probe AC impedance method from 100 Hz to 1 MHz (Hewlett-Packard, HP4194A) and the temperature controller of a Linkam LTS-E350 system. The AC impedance measurements under humidified conditions were conducted in the frequency range of 100 Hz to 1 MHz (Wayne Kerr Electronics, 6440B), and temperature and humidity were controlled using a compact constant-temperature and -humidity chamber (IW223, Yamato Scientific Co., Ltd.). Powder samples were pressed into pellets (3 mm in diameter), then coated with Ag paste on both sides and connected using gold wires (25 μm in diameter). The adsorption isotherms of H_2O vapor were measured at 298 K using a BELSORP-max II apparatus (MicrotracBEL), and nitrogen adsorption isotherms were measured at 77 K using the same instrument. Each sample was subjected to vacuum drying for pretreatment.

Synthesis and thermal stability of ImTP (Scheme S1). A mixture of octaaminotetraphenylene hydrochloride (101.2 mg, 141.3 μmol), triethyl orthoformate (167.6 mg, 1.13 mmol) and $ZrCl_4$ (13.2 mg 56.6 μmol) in dry MeOH (4 mL) was stirred at room temperature for 4 h. After completion of the reaction, MeOH was removed under reduced pressure. The residue was washed three times with EtOH. The resulting wet cake was dissolved in 20 mL water and $NaHCO_3$ (190.0 mg, 2.26 mmol) was added to the

solution. The solution immediately turned cloudy, and solid precipitate appeared. The precipitate was filtered and washed with water three times. The solid was dried under vacuum to give 58 mg (yield 88%). ImTP (2 mg) was dissolved in EtOH (3 mL) and diluted with diethyl ether (Et_2O) by vapor diffusion to give yellow single crystals of **ImTP**•(Et_2O)•3(H_2O) in 2−3 days. Single crystals containing Et_2O were unstable at room temperature, and solvent molecules easily removed, causing changes in formula of the crystal. Elemental analysis was performed using crystalline samples after drying under vaccum at 298 K, followed by storing in air at 298 K. Elemental analysis for **ImTP**•4(H_2O). Calcd for $C_{28}H_{16}N_8$•4(H_2O): C, 62.68; H, 4.51; N, 20.88. Found C, 62.52; H, 4.51; N, 20.82. ^{1}H NMR (400 MHz, DMSO-d_6) δ 12.37 (s, 4H), 8.16 (s, 4H), 7.25-7.36 (br d, 8H).

Preparation of H_nImTP^{n+}Cl^-_n. In a Schlenk flask (50 mL) **ImTP** (50.0 mg, 107.6 μmol) was dissolved in EtOH (10 mL). Hydrochloric acid (35–37%, 109.3 μL × n, 113.0 μmol × n) was added according to the value of n (the number of proton). Then the mixture was stirred at room temperature for 1 h. After solvent was removed under reduce pressure, the solid was washed with water (for **H_1ImTP$^+$**Cl$^-$ and **H_2ImTP^{2+}**Cl$^-_2$) or EtOH (for **H_4ImTP^{4+}**Cl$^-_4$). Single crystals of **H_nImTP^{n+}Cl^-_n** were prepared as follows. **H_1ImTP$^+$**Cl$^-$ (2 mg) was dissolved in EtOH (3 mL) and diluted by $CHCl_3$ by vapor diffusion to give brown single-crystals of **H_1ImTP$^+$Cl$^-$**•2($CHCl_3$) in 5 – 7 days. **H_2ImTP^{2+}**Cl$^-_2$ (2 mg) was dissolved in EtOH (3 mL) and diluted with tetrahydrofuran (THF) by vapor diffusion to give brown single-crystals of **H_2ImTP^{2+}Cl$^-_2$**•4(THF) in 2 weeks. Obtained single crystals were unstable at room temperature, and solvent molecules easily removed, causing changes in formula of the crystal. Elemental analysis was performed using crystalline samples after drying under vacuum at 298 K, followed by storing in air at 298 K. Elemental analysis of **H_1ImTP$^+$Cl$^-$**. Calcd for $C_{28}H_{17}N_8Cl$•3.3(H_2O): C, 60.01; H, 4.24; N, 20.00. Found C, 60.16; H, 4.12; N, 19.72. ^{1}H NMR (400 MHz, DMSO-d_6) δ 8.58 (s, 4H), 7.43 (s, 8H). **H_2ImTP^{2+}**Cl$^-_2$. Calcd for $C_{28}H_{18}N_8Cl_2$•3.5(H_2O): C, 52.80; H, 4.11; N, 17.59. Found C, 53.02; H, 3.98;

N, 17.32. ^{1}H NMR (400 MHz, DMSO-d_6) δ 8.84 (s, 4H), 7.51 (s, 8H). It was not possible to obtain single crystals of **H$_4$ImTP^{4+}**Cl^{-}_4. Elemental analysis was performed using powder samples vacuum-dried at 298 K. **H$_4$ImTP^{4+}**Cl^{-}_4. Calcd for $C_{28}H_{20}N_8Cl_4$•5.5(H_2O): C, 47.41; H, 4.40; N, 15.80. Found C, 47.75; H, 4.08; N, 15.46. ^{1}H NMR (400 MHz, DMSO-d_6) δ 9.20 (s, 4H), 7.62 (s, 8H).

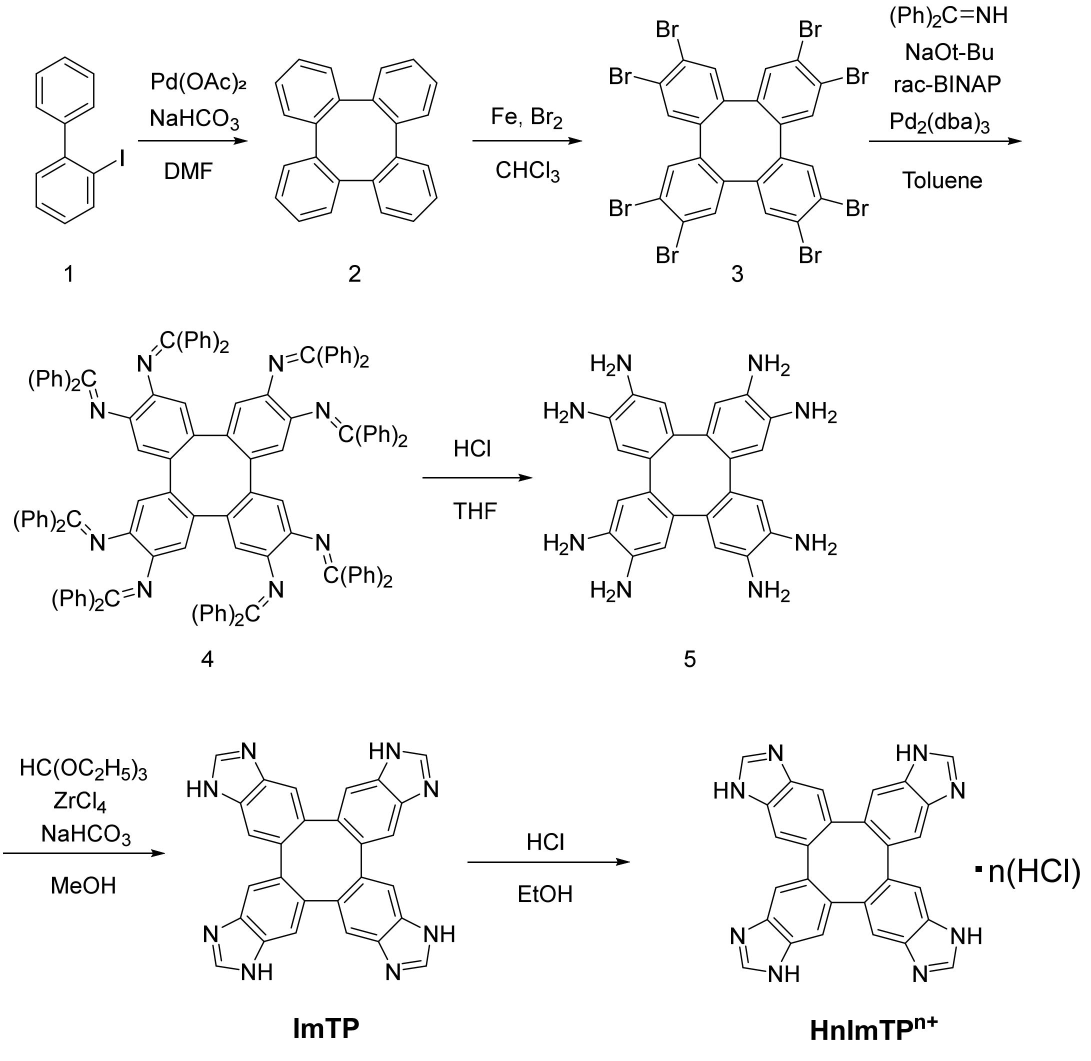

Scheme S1. Synthetic route of **ImTP** and **H$_n$ImTP^{n+}**Cl^{-}_n.

Crystal structural determination. Crystallographic data of **ImTP**•(Et_2O)•3(H_2O), **H$_1$ImTP^{+}**Cl^{-}•2 ($CHCl_3$), and **H$_2$ImTP^{2+}**Cl^{-}_2•4(THF) were collected using a Rigaku RAPID-II diffractometer equipped

with a rotating anode fitted with a multilayer confocal optic and using Cu K_α (λ = 1.54187 Å) radiation from a graphite monochromator (Table S1). Structural refinements were performed using the full-matrix least-squares method on F^2. Calculations were performed using Crystal Structure software packages.[51] All the parameters, except for those of the hydrogen atoms, were refined using anisotropic temperature factors.

Table S1. **Crystal data, data collection, and reduction parameters for H_nImTP^{n+}Cl^-_n.**

**Crystal**	**ImTP**•(Et_2O)•3(H_2O)	**H_1ImTP$^+$Cl$^-$**•2($CHCl_3$)	**H_2ImTP^{2+}Cl$^-_2$**•4(THF)
Chemical formula	$C_{28}H_{16}N_8$•($C_4H_{10}O$)3(H_2O)	$C_{28}H_{17}N_8Cl$•2($CHCl_3$)	$C_{28}H_{18}N_8Cl_2$•4(C_4H_8O)
Formula weight	589.61	739.68	825.82
T, K	100	100	100
Space group	*C*2/*c*	*C*2/*c*	*I*-42*d*
a, Å	16.0551(7)	15.1319(8)	12.3282(19)
b, Å	13.2714(6)	13.1893(6)	12.3282(19)
c, Å	16.4311(8)	16.3571(8)	28.108(2)
α, deg	–	–	–
β, deg	106.421(7)	102.978(7)	–
γ, deg	–	–	–
V, Å^3	3358.23	3181.15	4271.98
Z	4	4	4
D_{calc}, g·cm^{-3}	1.160	1.544	1.284
μ, cm^{-1}	0.654	6.007	1.785
Reflections measured	17906	17992	23393
Independent reflections	3060	2887	1941
Reflections used	3060	2887	1941
R_{int}	0.0871	0.0926	0.1331

R_1 [a]	0.0908	0.0853	0.0839
R_{all}	0.2688	0.2082	0.1853
$R_w(F_2)$ [a]	0.2977	0.2426	0.2257
GOF	0.971	1.199	0.935
CCDC	2472294	2472296	2472295

[a] $R_1 = \Sigma||F_o| - |F_c|| / \Sigma |F_o|$ and $R_w = (\Sigma\omega(|F_o| - |F_c|)^2 / \Sigma\omega F_o^2)^{1/2}$.

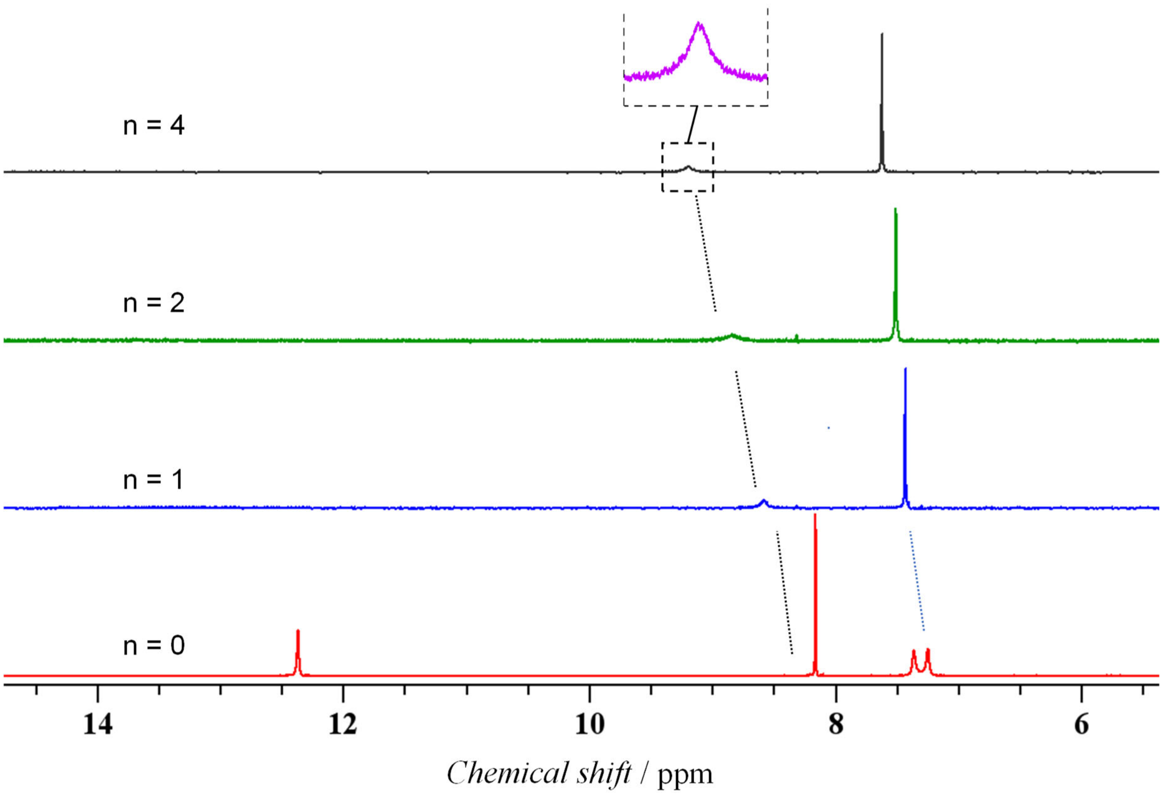

Figure S1. The *n*-dependent ^{1}H NMR spectra in DMSO-d_6.

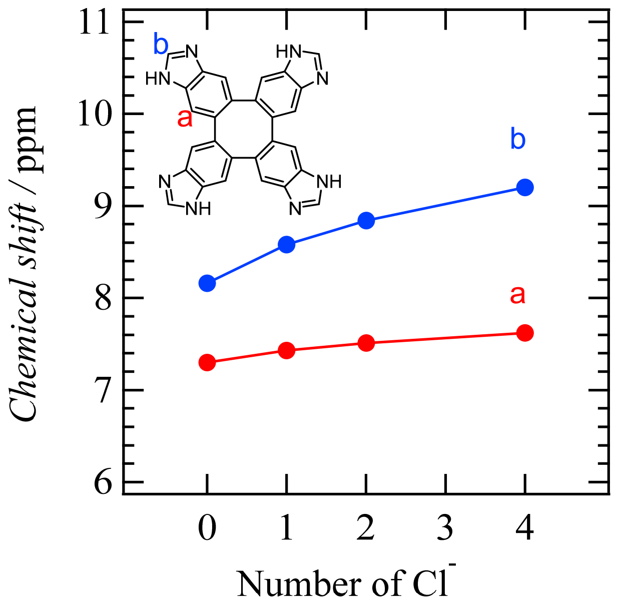

Figure S2. The *n*-depedent chemical shifts for H_a and H_b protons.

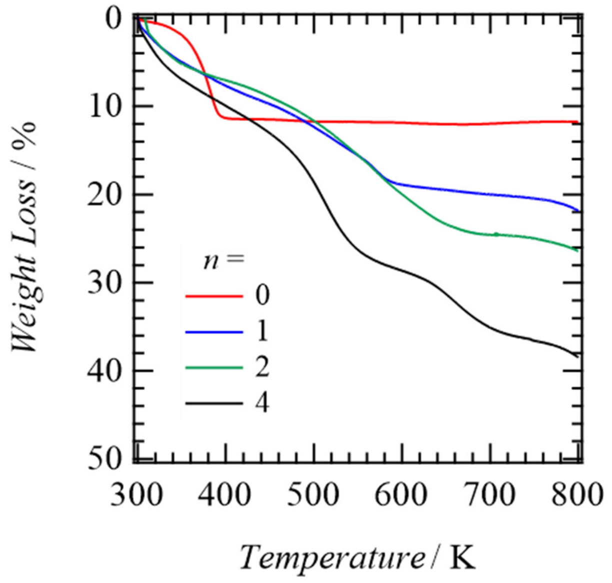

Figure S3. TG charts of $\mathbf{H_{\mathit{n}}ImTP^{\mathit{n}+}}Cl^{-}{}_{n}{\bullet}x(H_2O)$. Powder samples were ovtained by vacuum-dried at 298 K and left under the air.

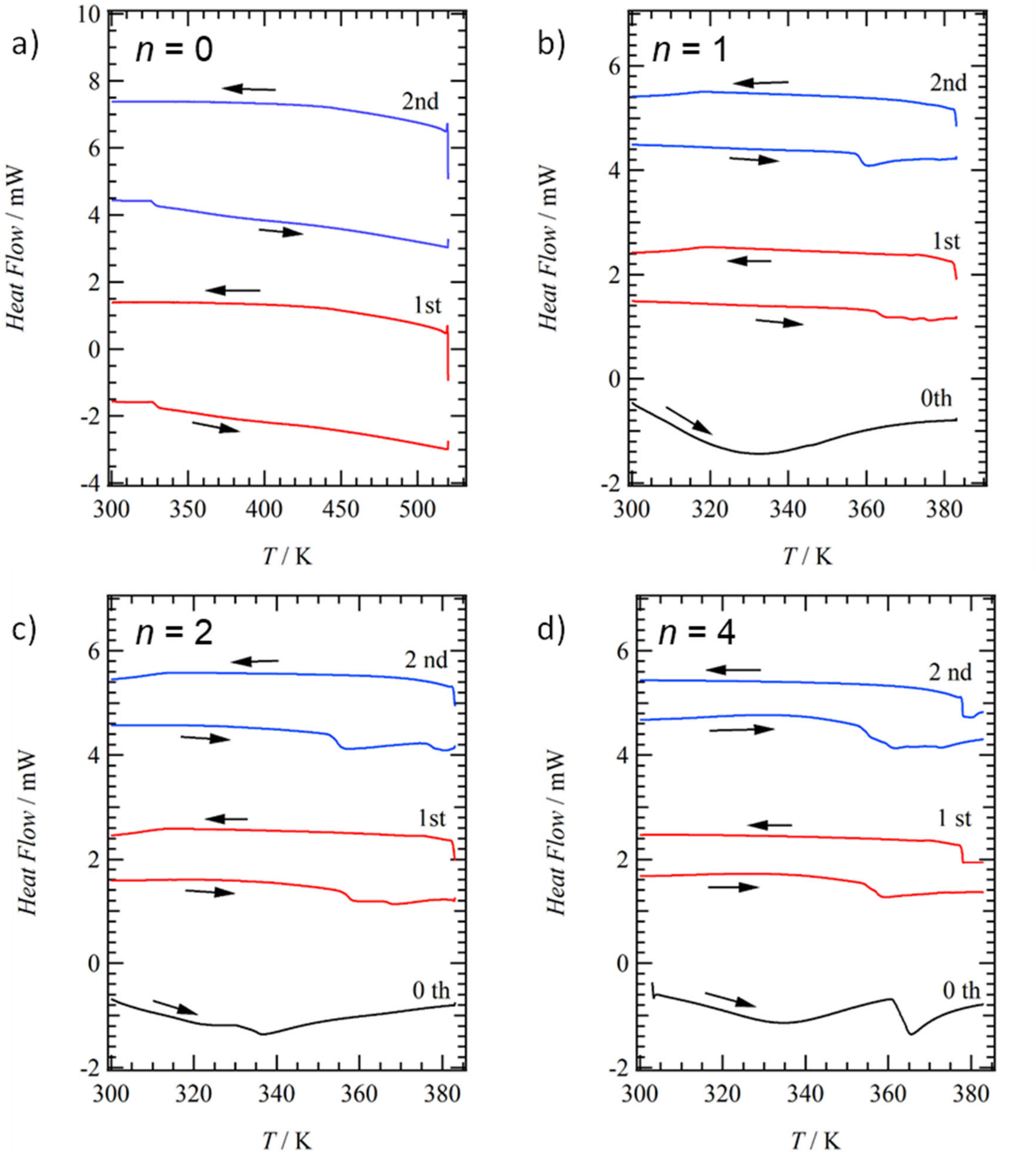

Figure S4. DSC charts of a) **ImTP**, b) **H_1ImTP$^+$**Cl^-•x(H_2O), c) **H_2ImTP^{2+}**$Cl^-{}_2$•x(H_2O), and d) **H_4ImTP$^+$**$Cl^-{}_4$•x(H_2O).

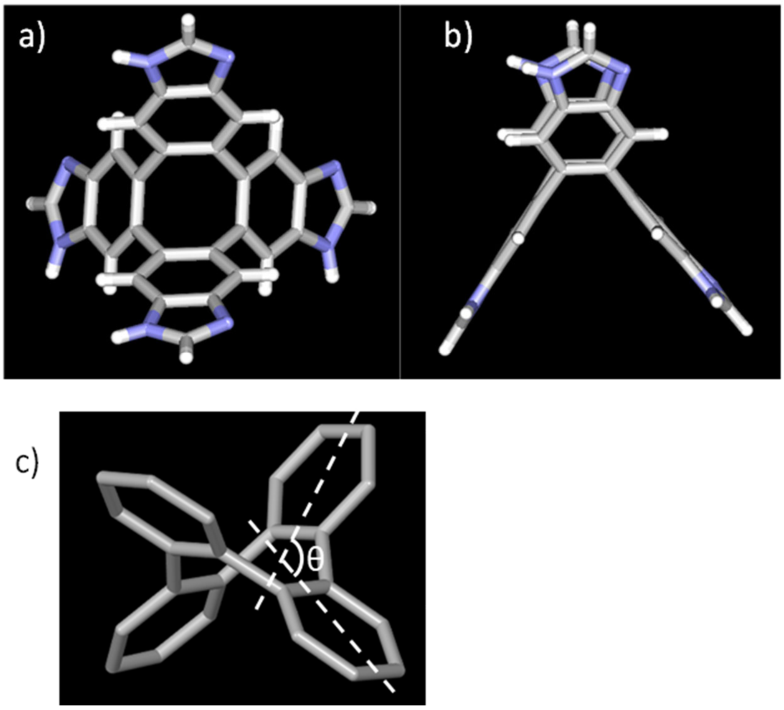

Figure S5. Saddle-shaped **ImTP** moelcule viewed along a) the normal diraction to the central cyclooctatetraene (COT) ring and b) the parallel to the COT ring. c) The angle between the neighboring benzene rings of **ImTP** unit.

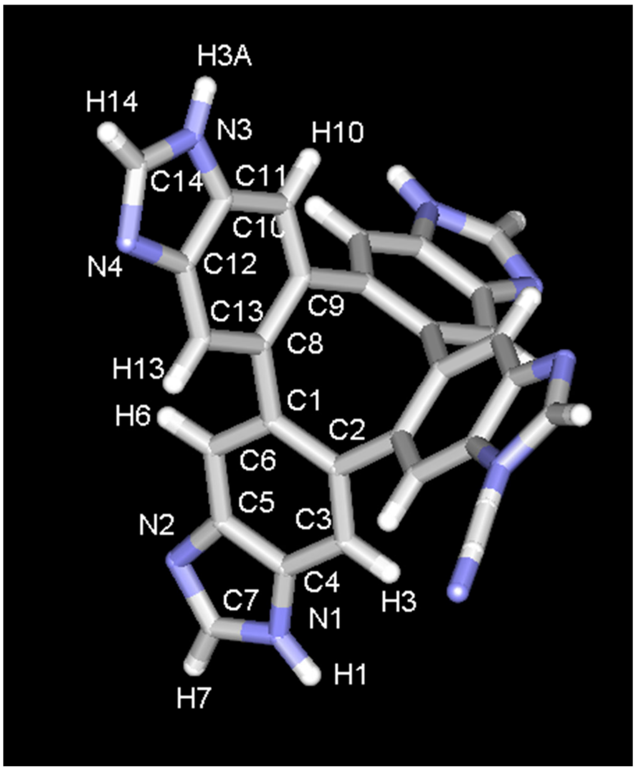

Figure S6. Crystallographically independent **ImTP** unit in **ImTP**•(Et_2O)•3(H_2O) singel crystal at T = 100 K.

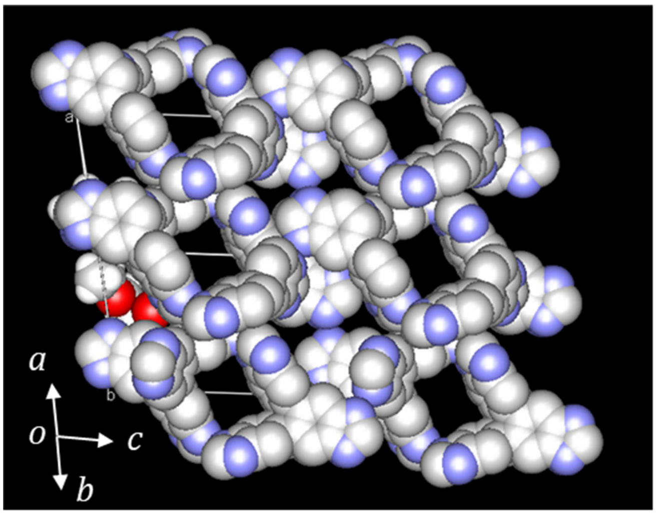

Figure S7. 1D channel along the a+*b*-axis surrounded by **ImTPs**. CPK representation without hydrogen atoms.

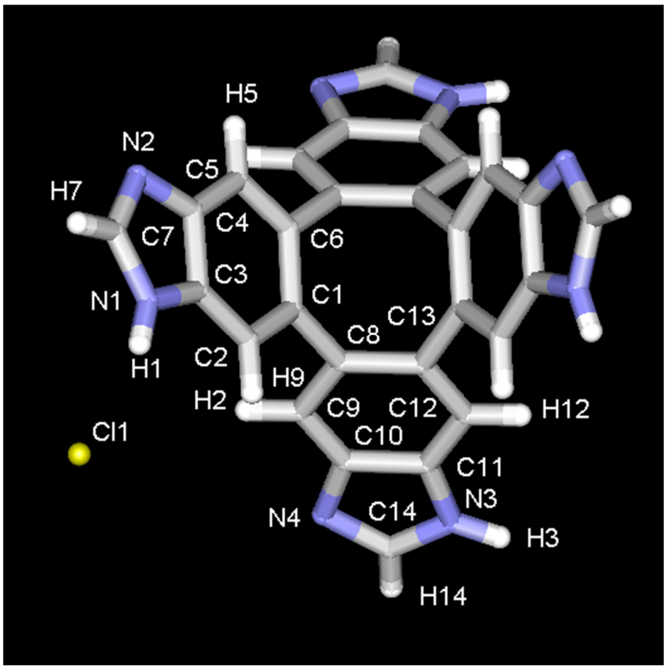

Figure S8. Crystallographically independent **H_1ImTP$^+$** unit in **H_1ImTP$^+$**Cl^-•2($CHCl_3$) singel crystal at T = 100 K.

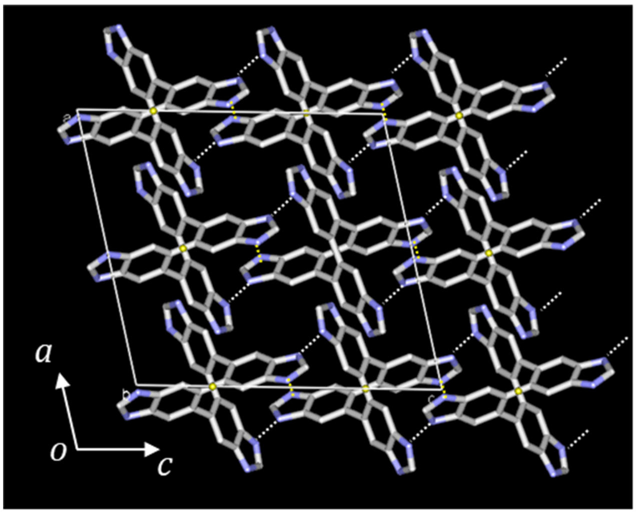

Figure S9. Unit cell of **H_1ImTP$^+$**Cl^-•2($CHCl_3$) viewed along the *b*-axis. Dashed lines indicate hydrogen bonds.

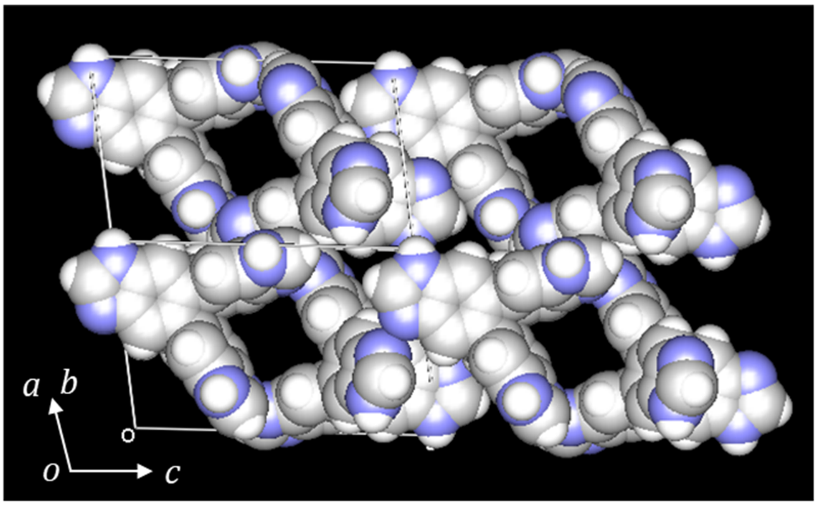

Figure S10. 1D channel along the *b*-axis surrounded by $\mathbf{H_1ImTP^+}$ cations in $\mathbf{H_1ImTP^+}Cl^-\cdot 2(CHCl_3)$.

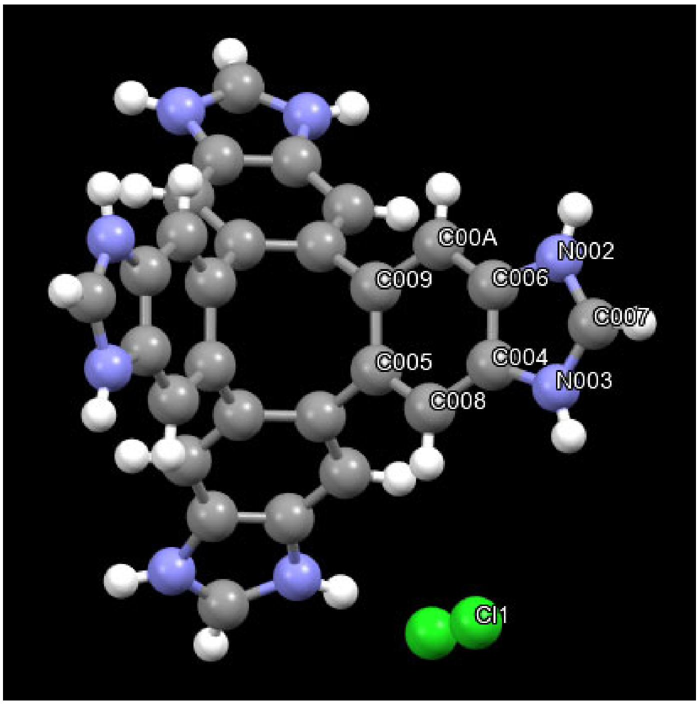

Figure S11. Crystallographically independent $\mathbf{H_2ImTP^{2+}}$ unit in $\mathbf{H_2ImTP^{2+}}Cl^-{}_2\cdot 4(THF)$ singel crystal at T = 100 K.

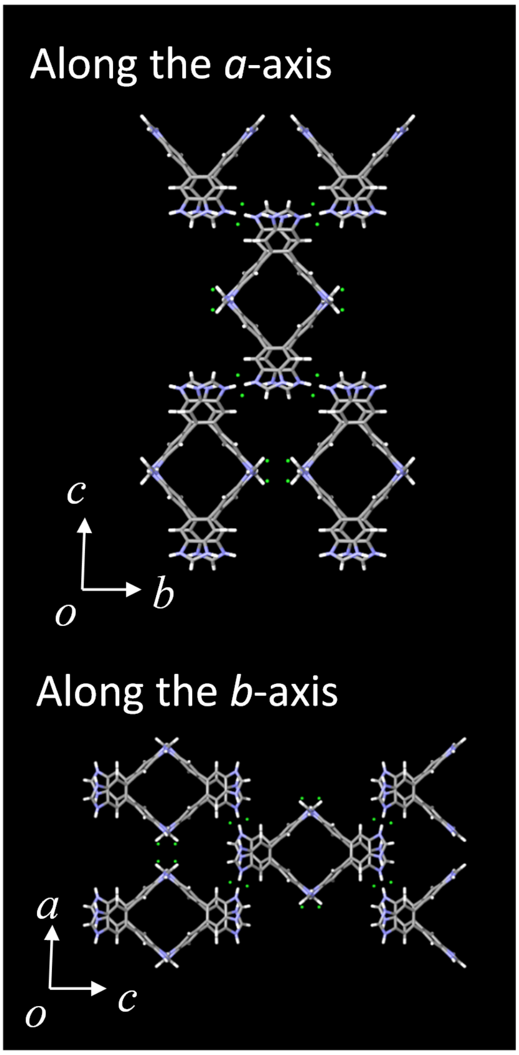

Figure S12. The two different directional 1D channel structures along the *a*-axis (upper) and along the *b*-axis (lower) in **H_2ImTP^{2+}**$Cl^{-}{}_2$•4(THF) singel crystal.

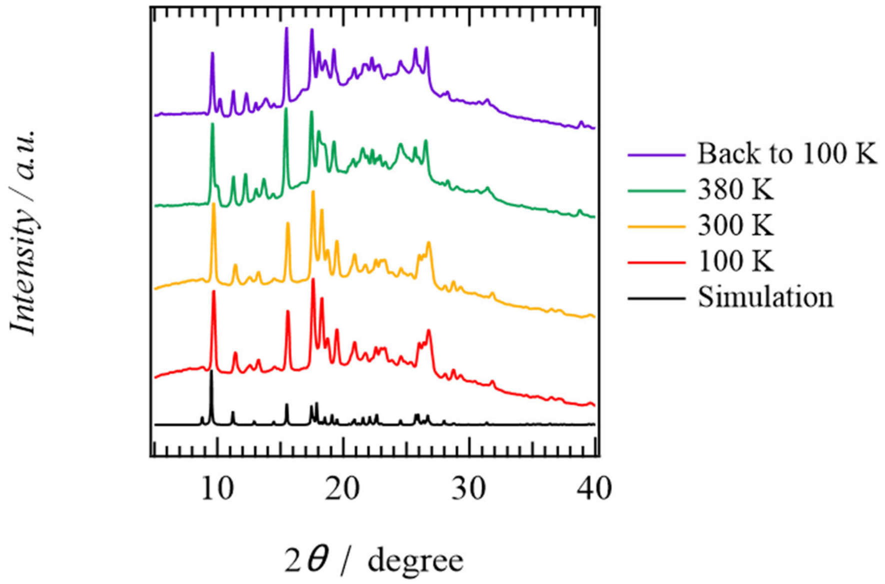

Figure S13. Temperature-dependent PXRD patterns of **ImTP** and the simulation pattern based on single-crystal X-ray structural analysis at 100 K.

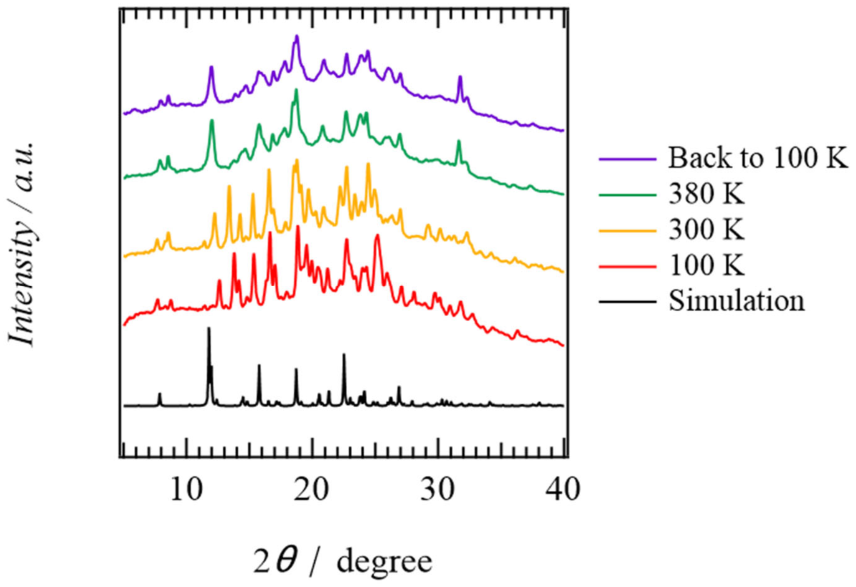

Figure S14. Temperature-dependent PXRD patterns of $\mathbf{H_2ImTP^{2+}}Cl^{-}{}_2$ and the simulation pattern based on single-crystal X-ray structural analysis at 100 K.

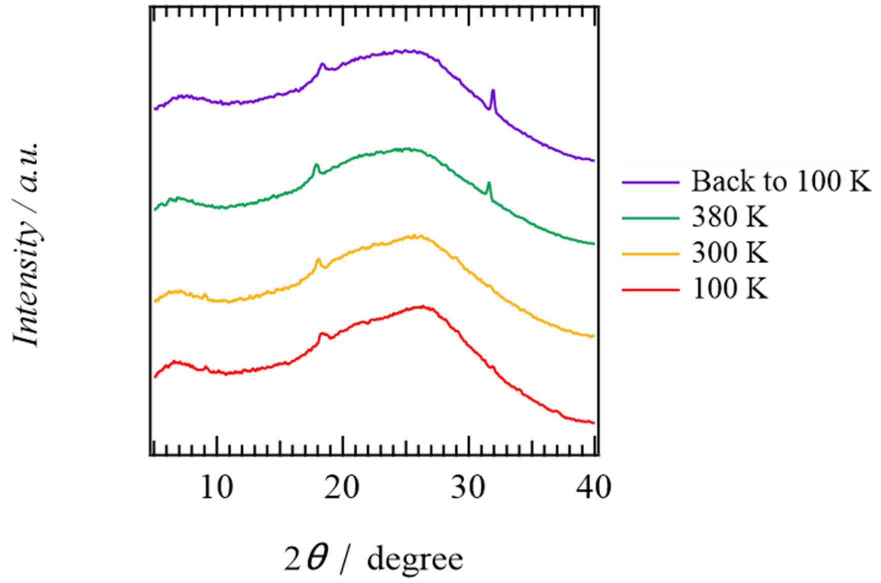

Figure S15. Temperature-dependent PXRD patterns of $\mathbf{H_4ImTP^{4+}}Cl^{-}{}_{4}$.

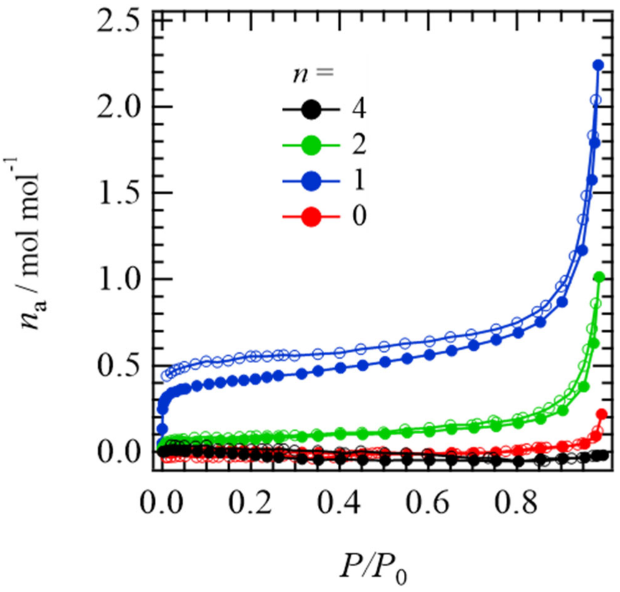

Figure S16. N_2 sorption isotherms of $\mathbf{H_{\mathit{n}}ImTP^{\mathit{n}+}}$•$Cl^{-}{}_{n}$ at 77 K. The n-dependent n_a vs. P/P_0 plots (n = 0, 1, 2, and 4).

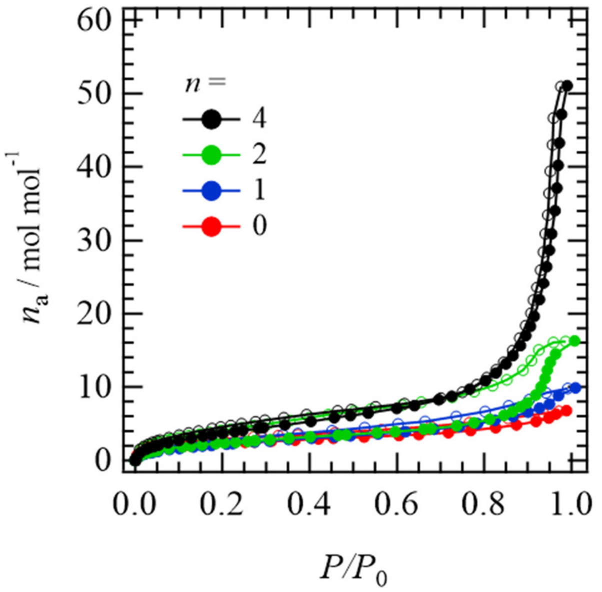

Figure S17. H_2O sorption isotherms of anhydrous $\mathbf{H}_n\mathbf{ImTP}^{n+}$•$Cl^-{}_n$ at 298 K. The n-dependent n_a vs. P/P_0 plots (n = 0, 1, 2, and 4).

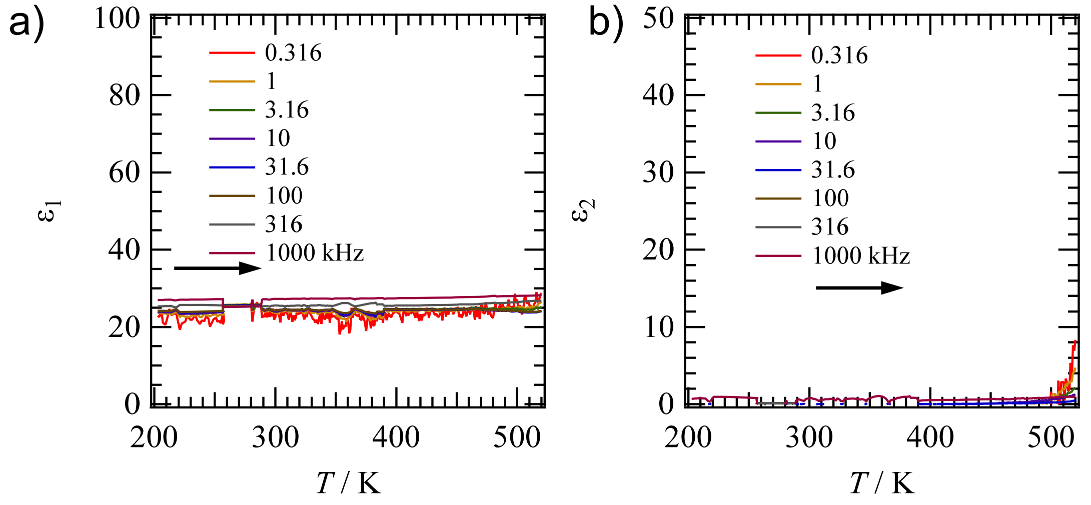

Figure S18. Temperature- and frequency-dependent a) ε_1 and b) ε_2 of **ImTP**•$x(H_2O)$ under RH = 0%.

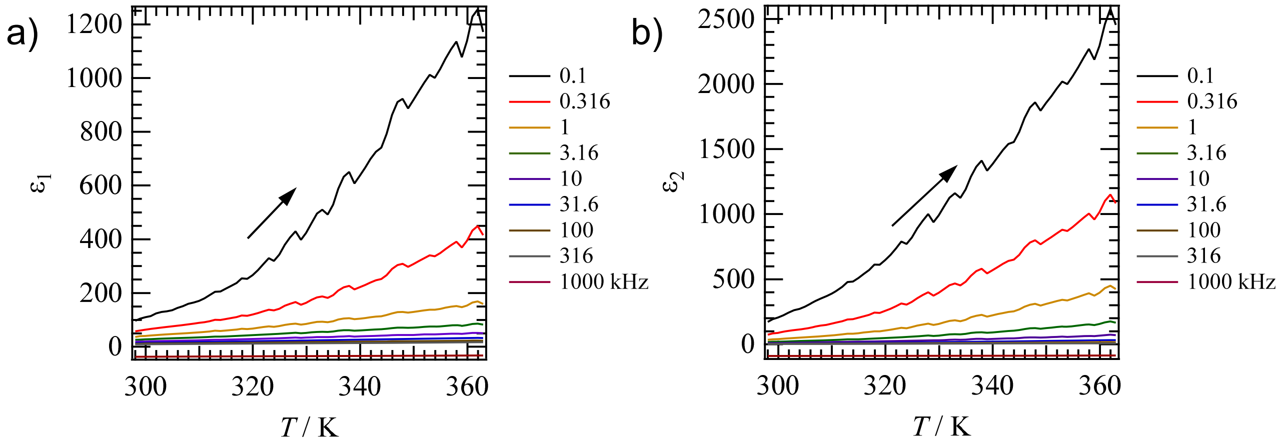

Figure S19. Temperature- and frequency-dependent a) ε_1 and b) ε_2 of **ImTP•**$x(H_2O)$ under RH = 40%.

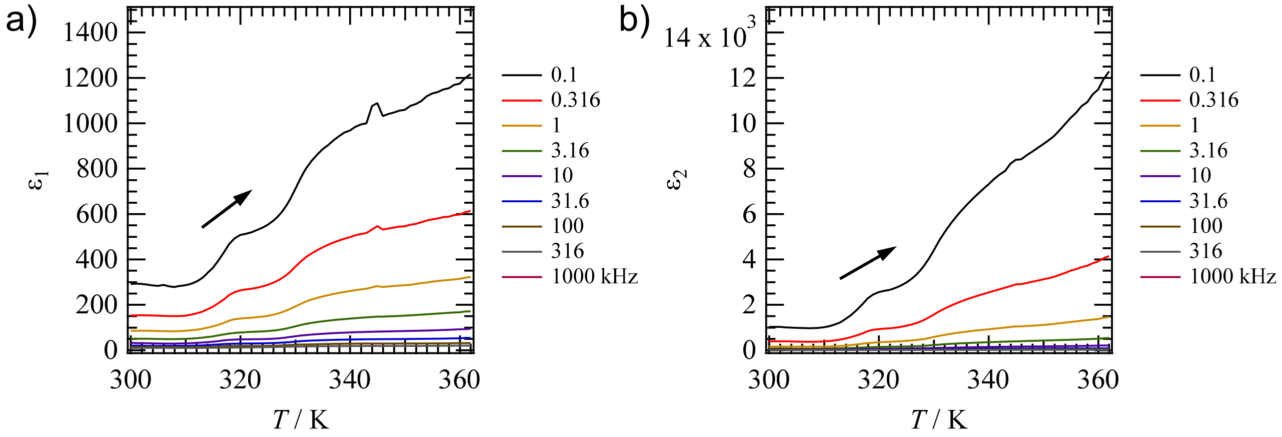

Figure S20. Temperature- and frequency-dependent a) ε_1 and b) ε_2 of **ImTP•**$x(H_2O)$ under RH = 98%.

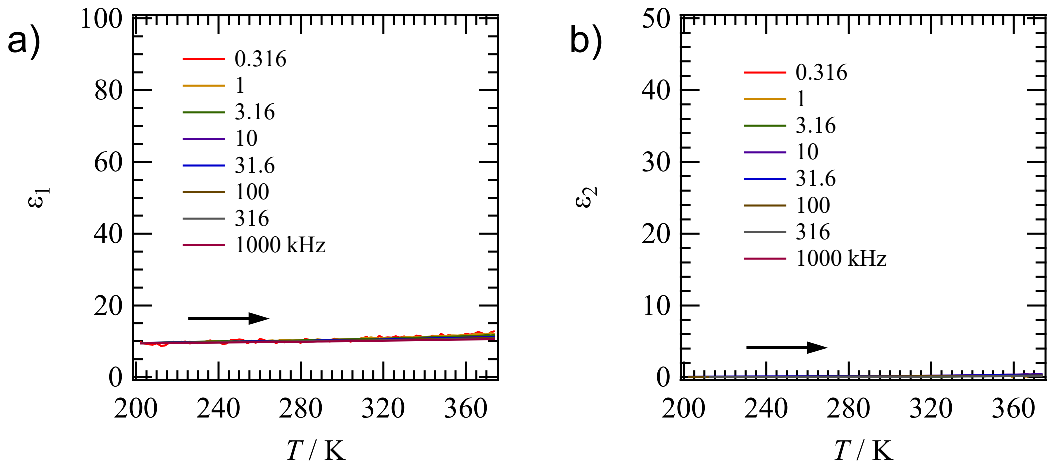

Figure S21. Temperature- and frequency-dependent a) ε_1 and b) ε_2 of **$H_1ImTP^+Cl^-$•**$x(H_2O)$ under RH = 0%.

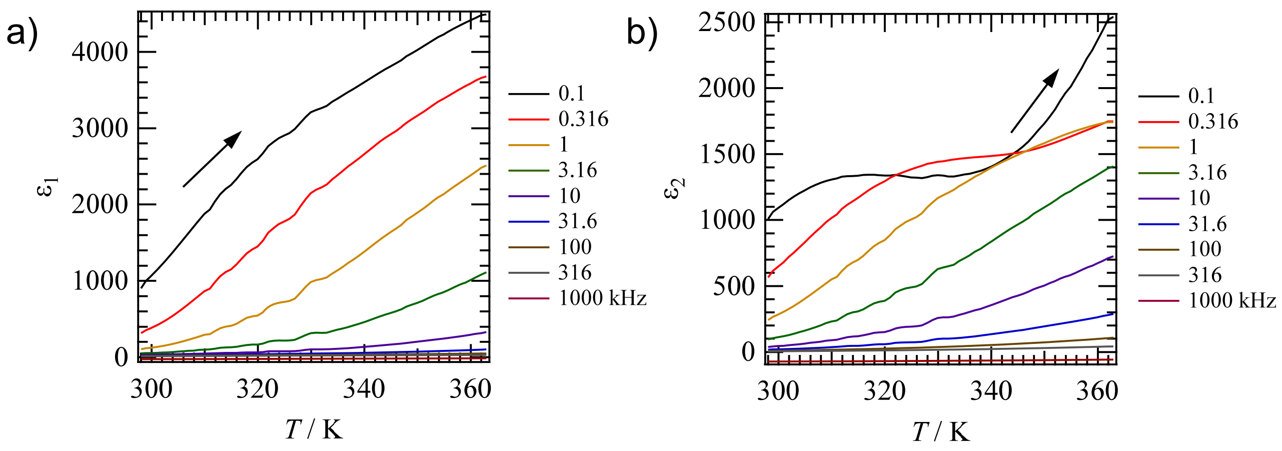

Figure S22. Temperature- and frequency-dependent a) ε_1 and b) ε_2 of **$H_1ImTP^+Cl^-$•**$x(H_2O)$ under RH = 40%.

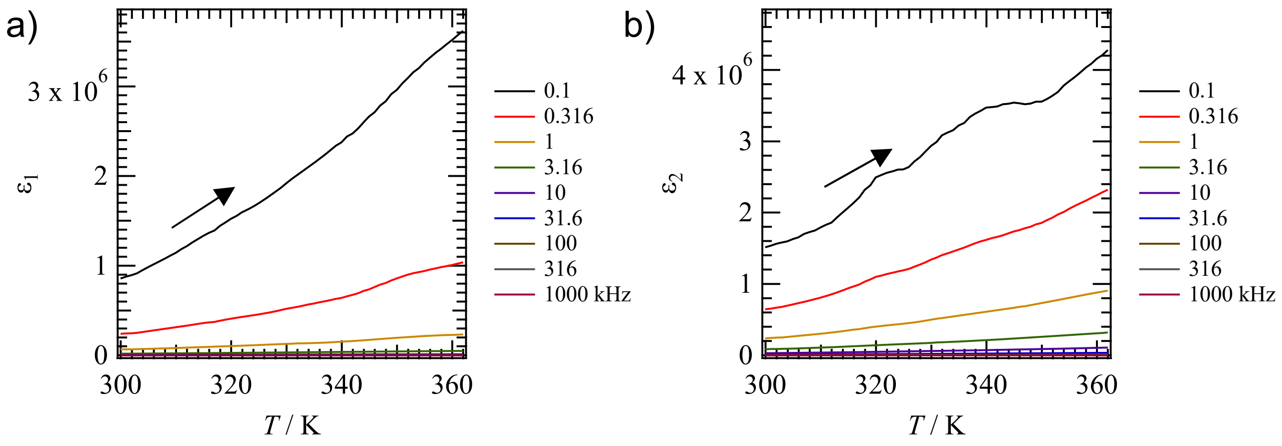

Figure S23. Temperature- and frequency-dependent a) ε_1 and b) ε_2 of **$H_1ImTP^+Cl^-$**$\bullet x(H_2O)$ under RH = 98%.

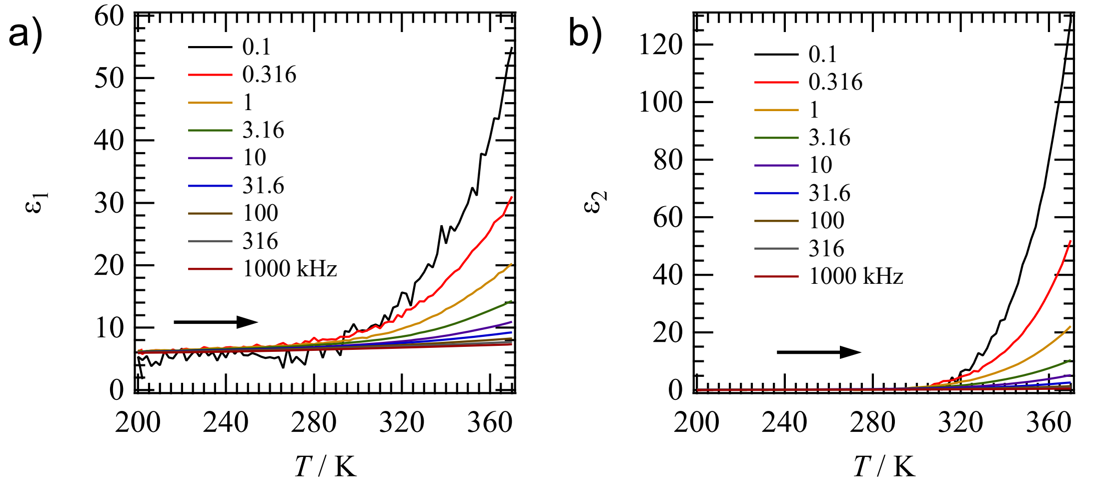

Figure S24. Temperature- and frequency-dependent a) ε_1 and b) ε_2 of **$H_2ImTP^{2+}Cl^-$**$_2\bullet x(H_2O)$ under RH = 0%.

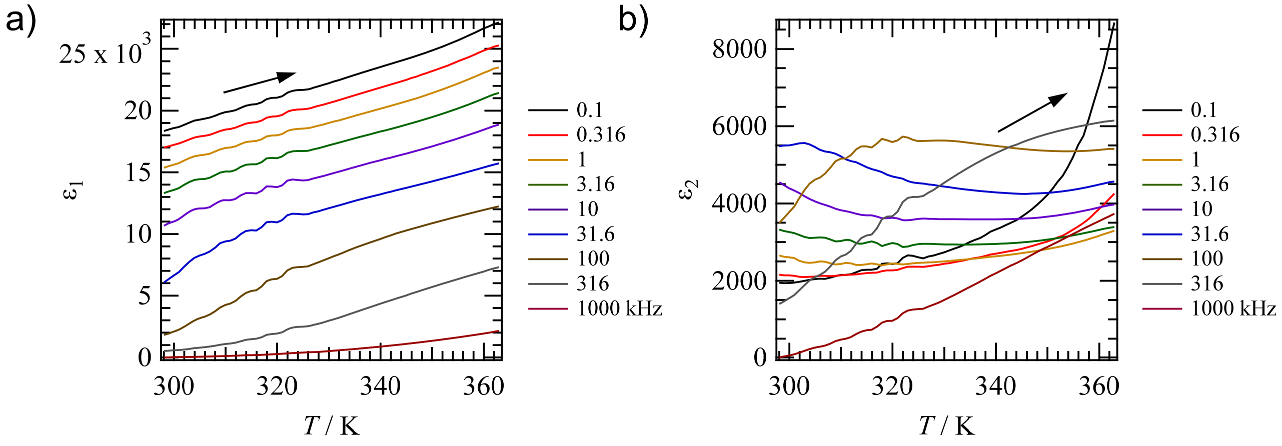

Figure S25. Temperature- and frequency-dependent a) ε_1 and b) ε_2 of **$H_2ImTP^{2+}Cl^{-}{}_2$**•x(H_2O) under RH = 40%.

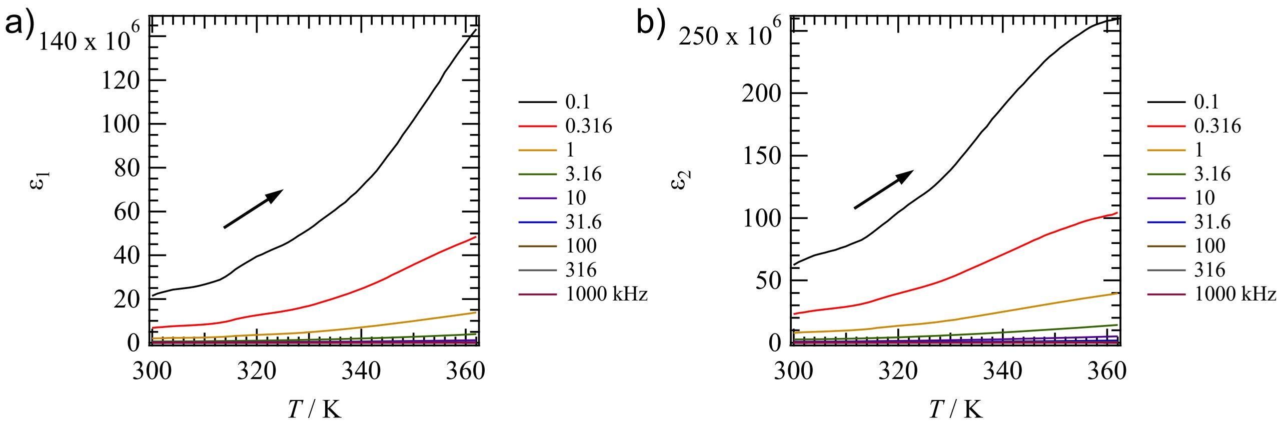

Figure S26. Temperature- and frequency-dependent a) ε_1 and b) ε_2 of **$H_2ImTP^{2+}Cl^{-}{}_2$**•x(H_2O) under RH = 98%.

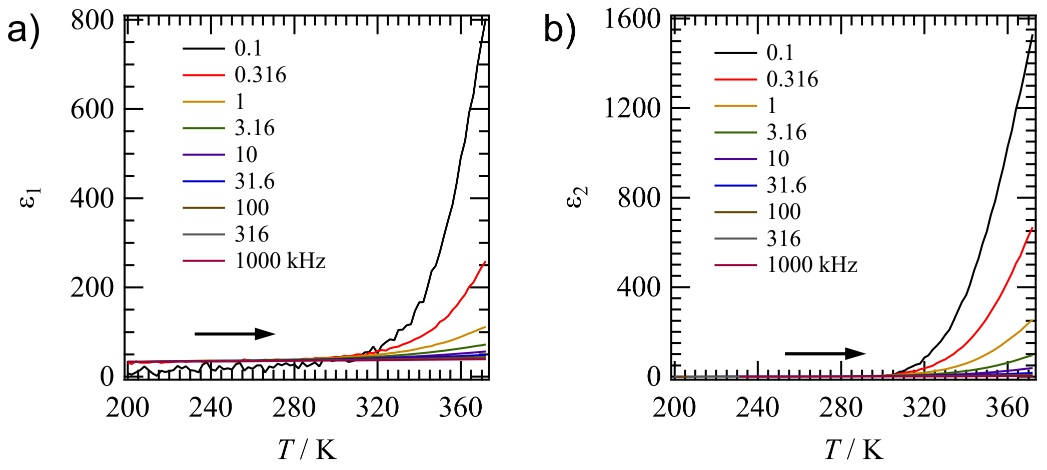

Figure S27. Temperature- and frequency-dependent a) ε_1 and b) ε_2 of **H_4ImTP^{4+}Cl$^-_4$**•x(H_2O) under RH = 0%.

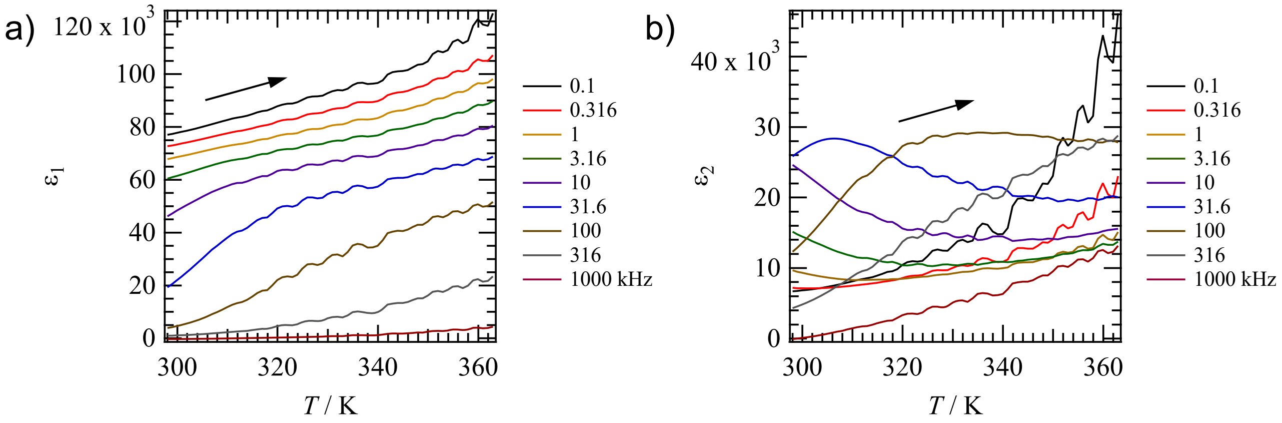

Figure S28. Temperature- and frequency-dependent a) ε_1 and b) ε_2 of **H_4ImTP^{4+}Cl$^-_4$**•x(H_2O) under RH = 40%.

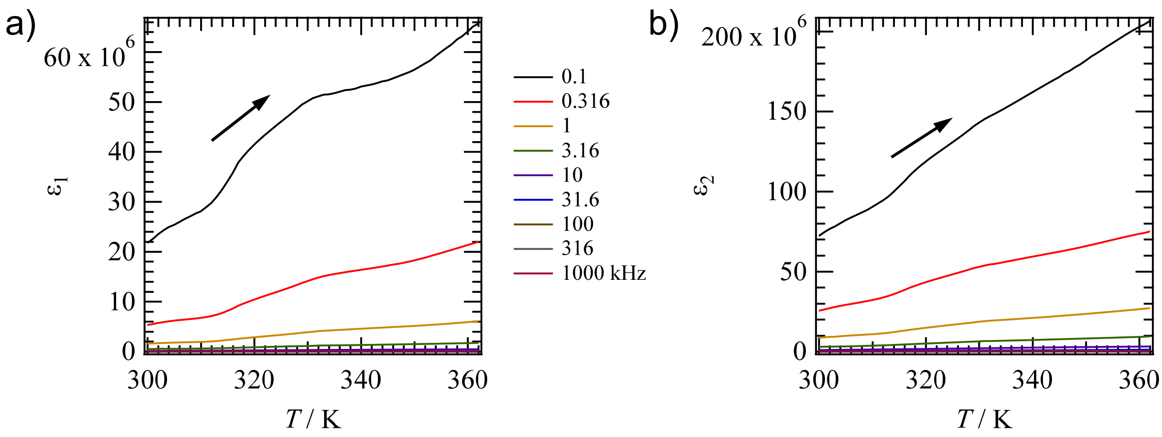

Figure S29. Temperature- and frequency-dependent a) ε_1 and b) ε_2 of **H_4ImTP^{4+}Cl$^-_4$**•x(H_2O) under RH = 98%.

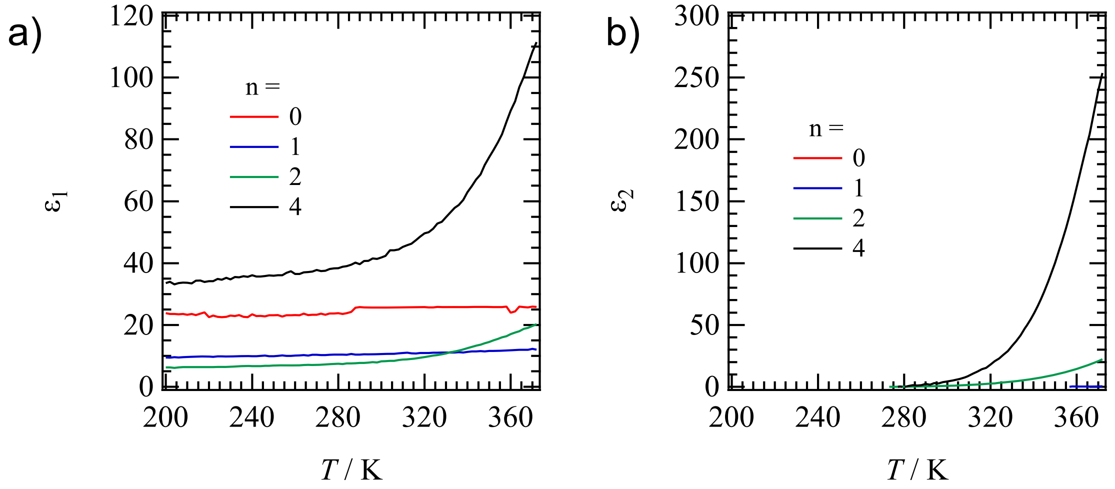

Figure S30. Temperature-dependent a) ε_1 and b) ε_2 of **H_nImTP^{n+}Cl^-_n**•x(H_2O) under f = 1 kHz and RH = 0%.

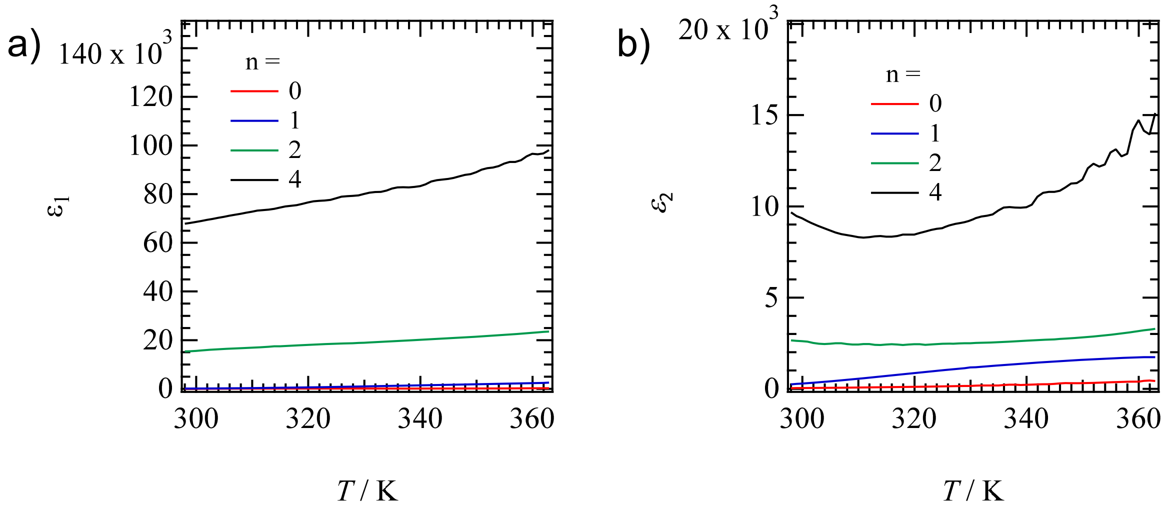

Figure S31. Temperature-dependent a) ε_1 and b) ε_2 of **H_nImTP^{n+}Cl$^{-}_{n}$•**$x(H_2O)$ under f = 1 kHz and RH = 40%.

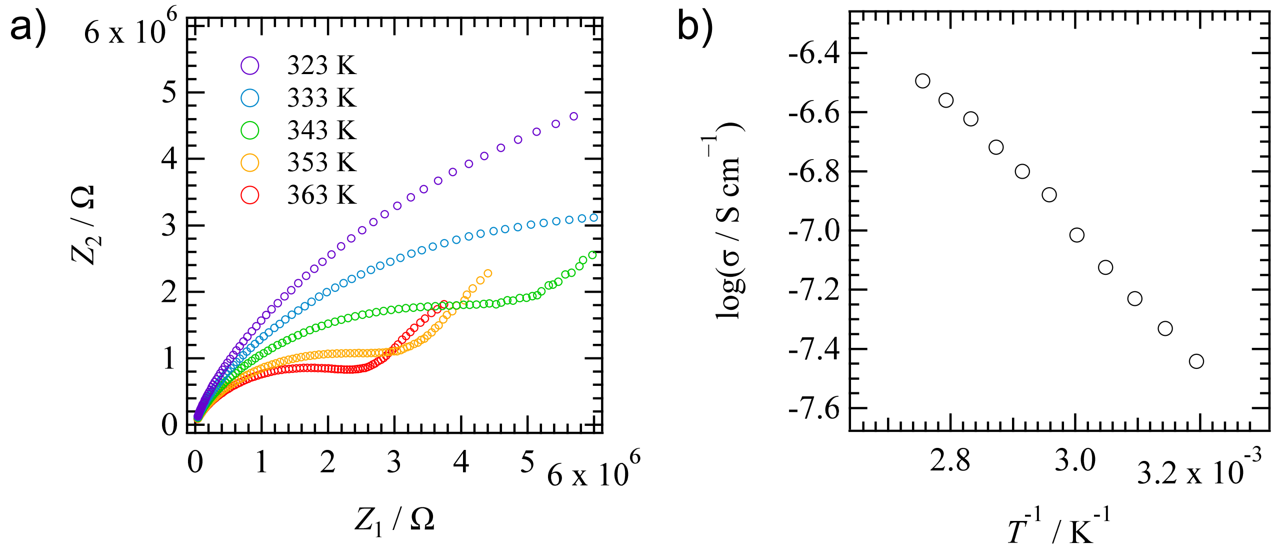

Figure S32. Temperature-dependent a) Nyquist plots and b) log σ_{H+} – T^{-1} plots of **ImTP•**$x(H_2O)$ under RH = 40%.

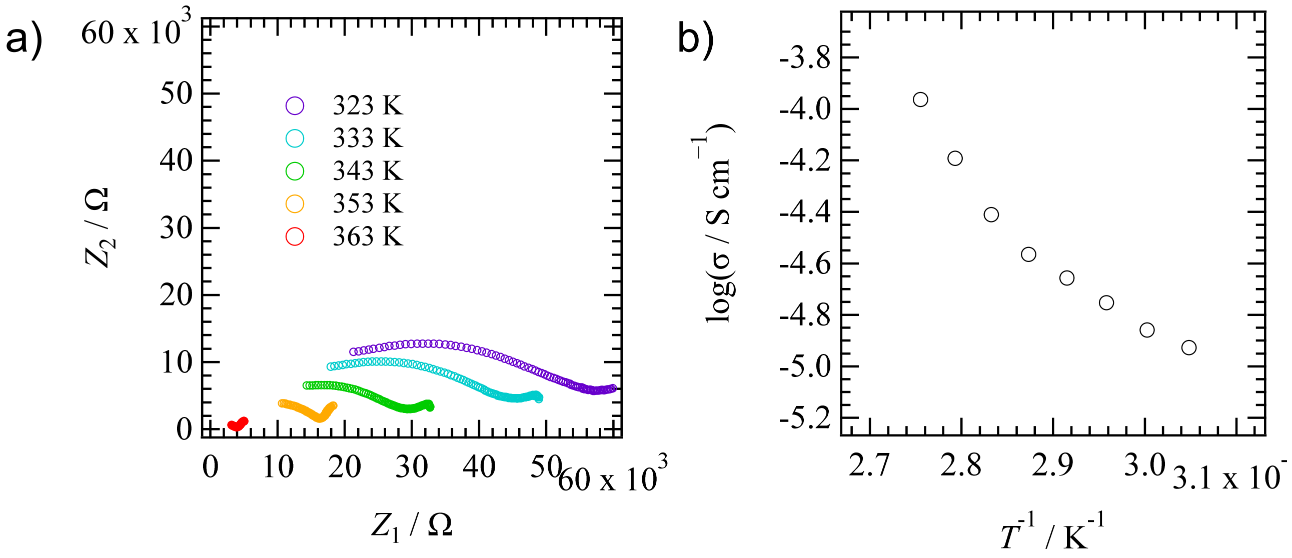

Figure S33. Temperature-dependent a) Nyquist plots and b) log σ_{H+} – T^{-1} plots of **ImTP•**$x(H_2O)$ under RH = 98%.

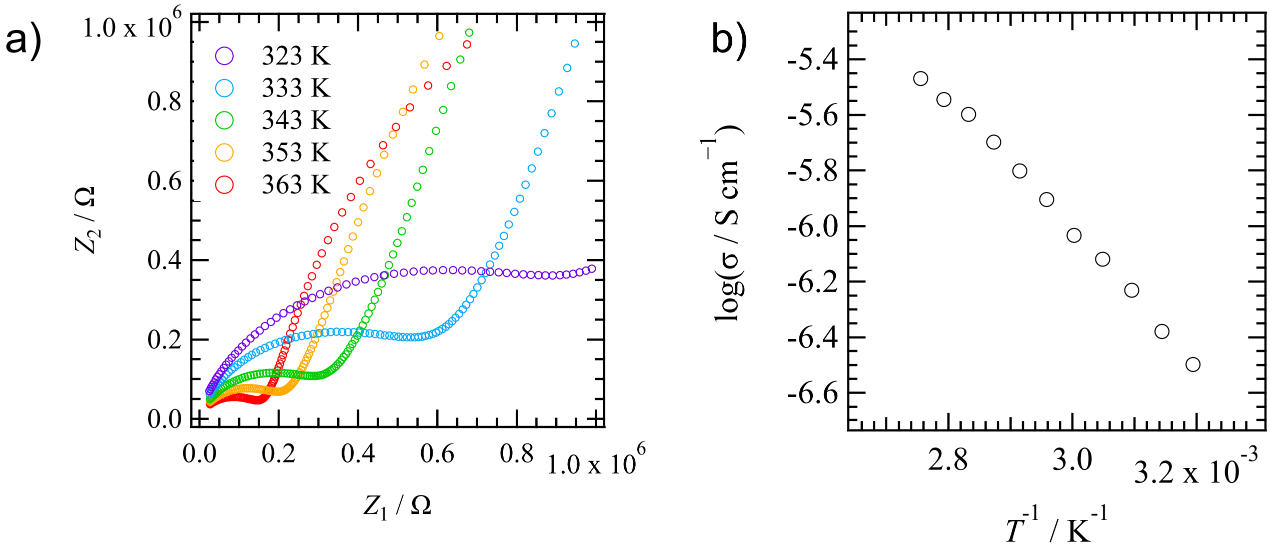

Figure S34. Temperature-dependent a) Nyquist plots and b) log σ_{H+} – T^{-1} plots of $\mathbf{H_1ImTP^+\ Cl^-}$**•**$x(H_2O)$ under RH = 40%.

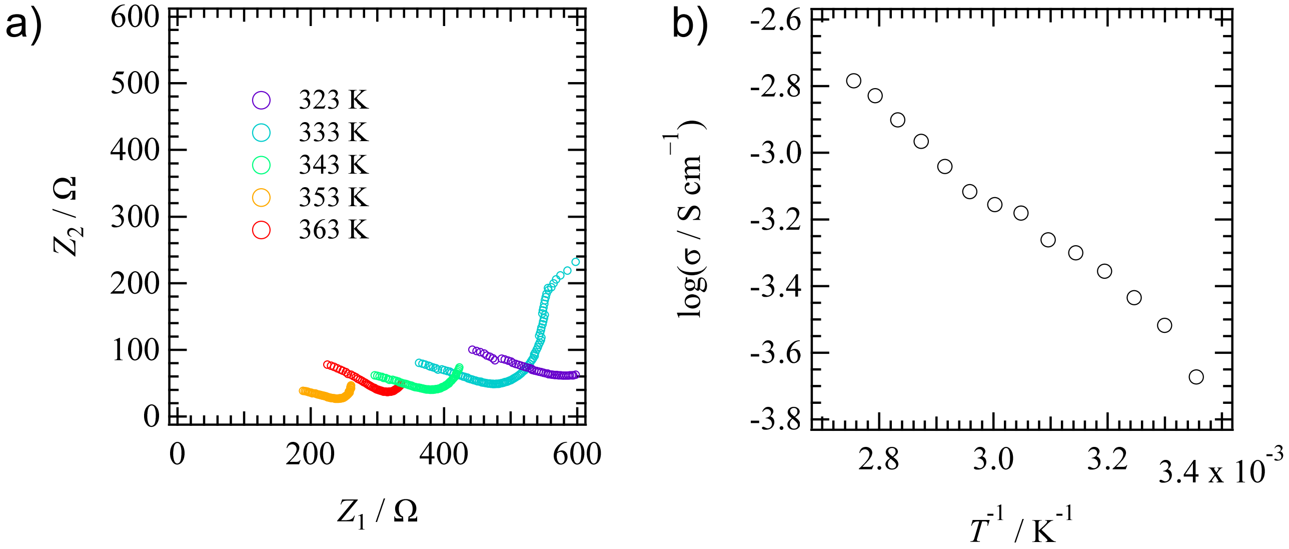

Figure S35. Temperature-dependent a) Nyquist plots and b) log σ_{H+} – T^{-1} plots of **H_1ImTP$^+$ Cl$^-$**•x(H_2O) under RH = 98%.

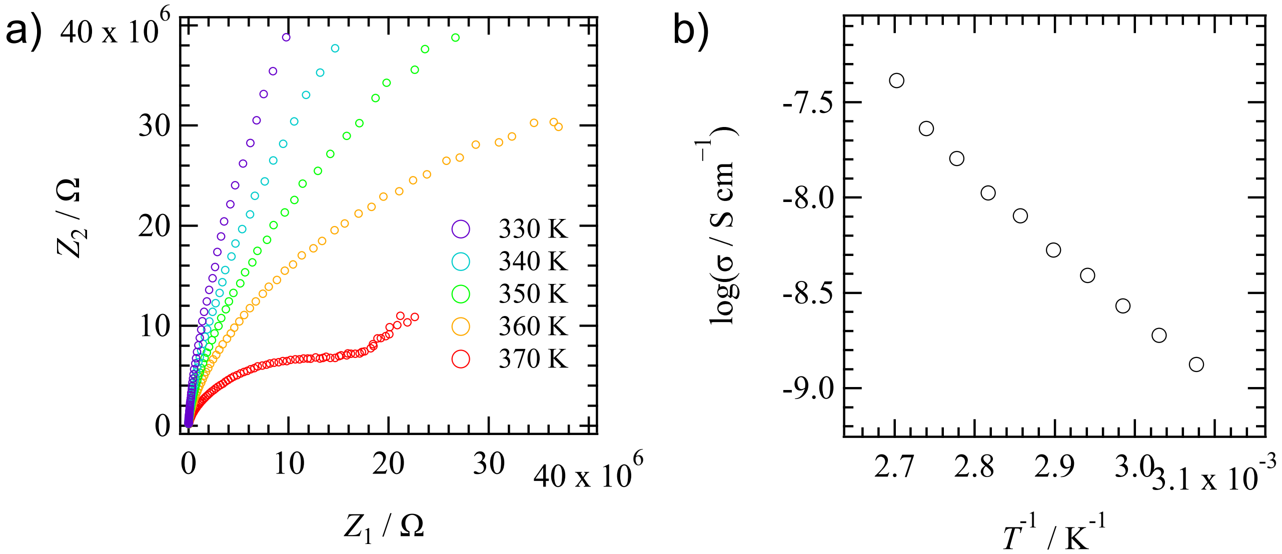

Figure S36. Temperature-dependent a) Nyquist plots and b) log σ_{H+} – T^{-1} plots of **H_2ImTP^{2+}Cl$^-_2$**•x(H_2O) under RH = 0%.

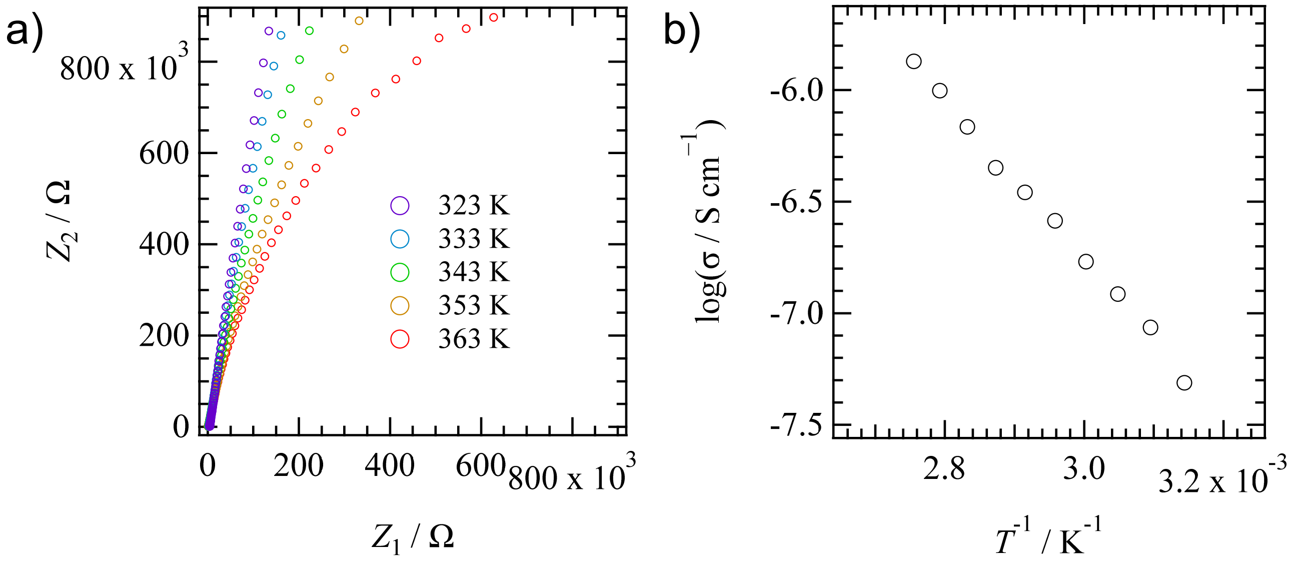

Figure S37. Temperature-dependent a) Nyquist plots and b) log $\sigma_{H+} - T^{-1}$ plots of $\mathbf{H_2ImTP^{2+}Cl^{-}_2}$•$x(H_2O)$ under RH = 40%.

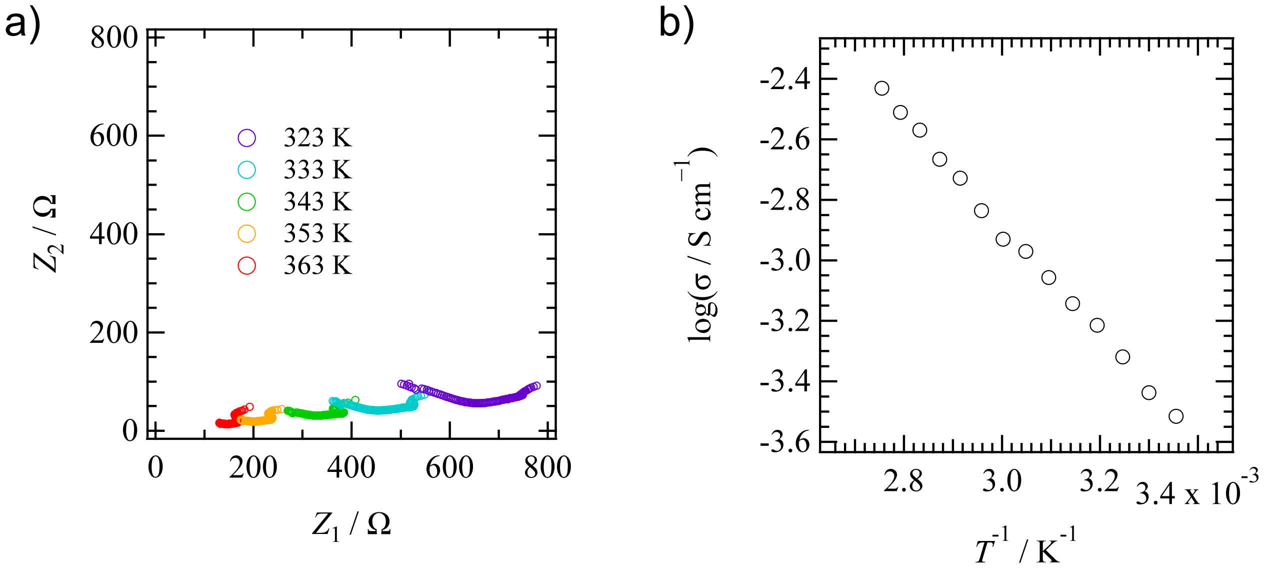

Figure S38. Temperature-dependent a) Nyquist plots and b) log $\sigma_{H+} - T^{-1}$ plots of $\mathbf{H_2ImTP^{2+}Cl^{-}_2}$•$x(H_2O)$ under RH = 98%.

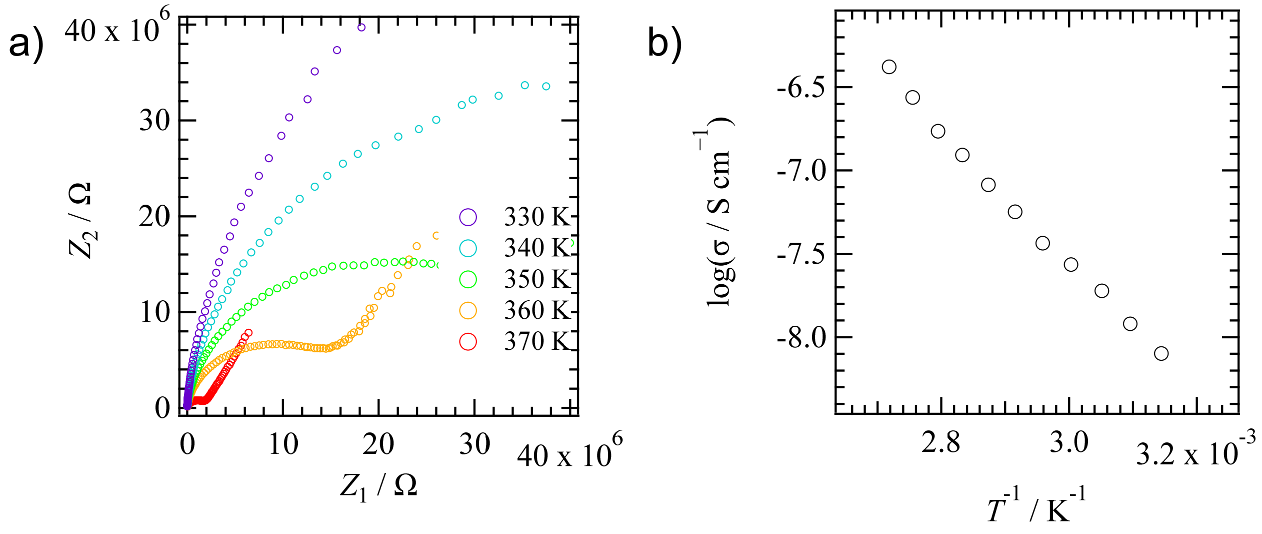

Figure S39. Temperature-dependent a) Nyquist plots and b) log σ_{H+} – T^{-1} plots of $\mathbf{H_4ImTP^{4+}Cl^-{}_4}$•$x$($H_2O$) under RH = 0%.

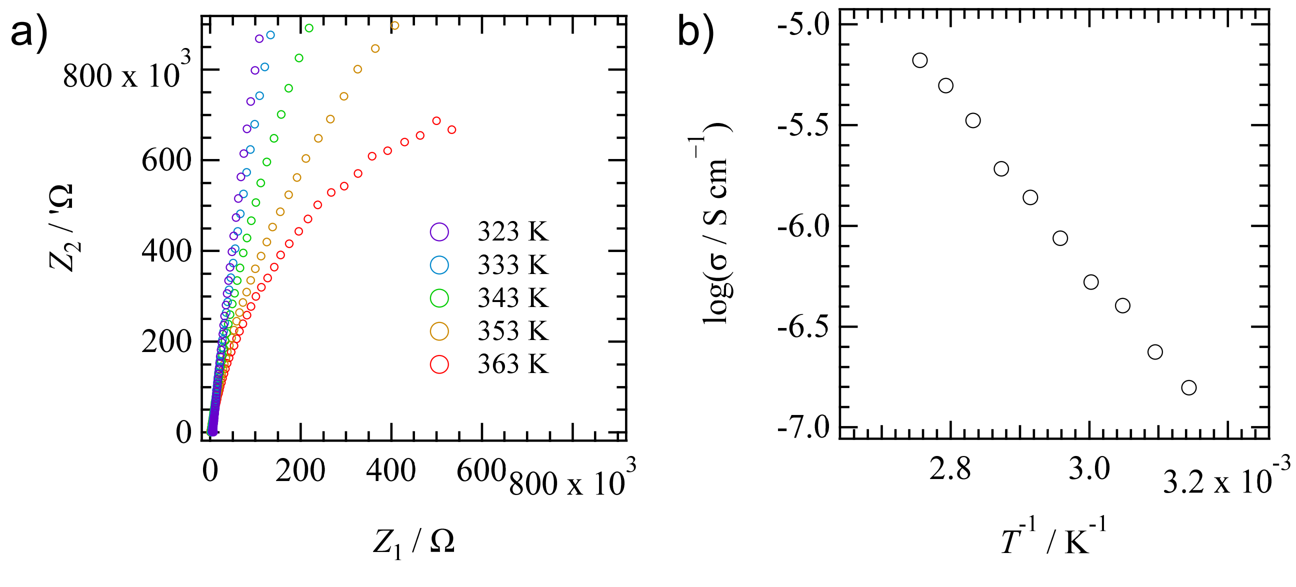

Figure S40. Temperature-dependent a) Nyquist plots and b) log σ_{H+} – T^{-1} plots of $\mathbf{H_4ImTP^{4+}Cl^-{}_4}$•$x$($H_2O$) under RH = 0%.

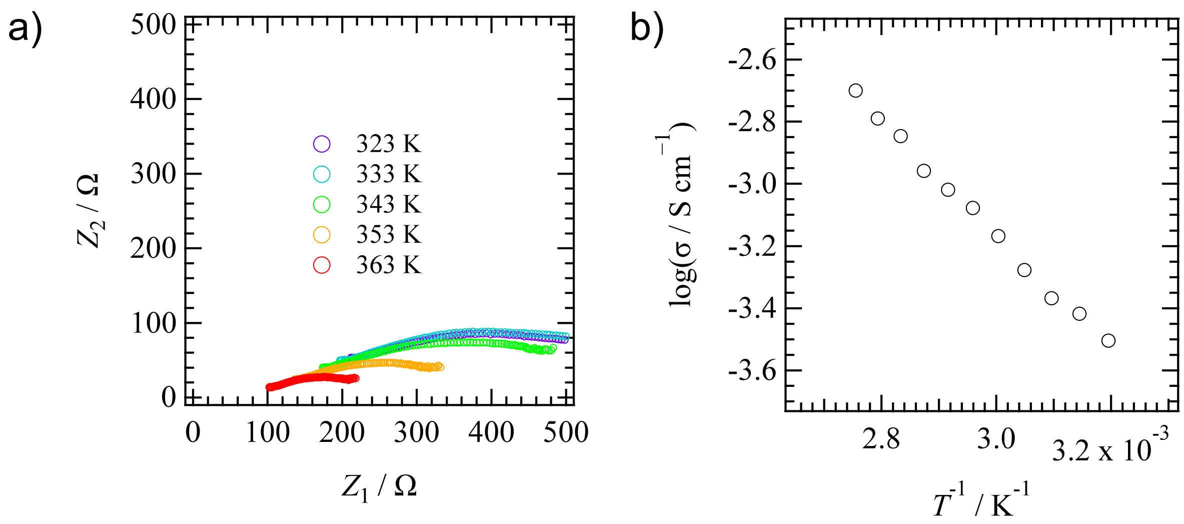

Figure S41. Temperature-dependent a) Nyquist plots and b) log σ_{H+} – T^{-1} plots of $\mathbf{H_4ImTP^{4+}Cl^{-}_4}$•$x(H_2O)$ under RH = 98%.
