# Supplementary material for: Proton Conductivities of Stepwise Protonated Imidazole‐Fused Tetraphenylene Derivatives
Source: Chemistry. 2025 Oct 9;31(62):e02622. doi: 10.1002/chem.202502622 (PMC12598376; doi:10.1002/chem.202502622)

checkCIF/PLATON report

Structure factors have been supplied for datablock(s) shelxt

THIS REPORT IS FOR GUIDANCE ONLY. IF USED AS PART OF A REVIEW PROCEDURE FOR PUBLICATION, IT SHOULD NOT REPLACE THE EXPERTISE OF AN EXPERIENCED CRYSTALLOGRAPHIC REFEREE.

No syntax errors found. CIF dictionary Interpreting this report

Datablock: shelxt

Bond precision: C-C = 0.0054 A Wavelength=1.54187

Cell:	a=16.0551(7)	b=13.2714(6)	c=16.4311(8)
	alpha=90	beta=106.421(7)	gamma=90
Temperature:	100 K		

	Calculated	Reported
Volume	3358.2(3)	3358.2(3)
Space group	C 2/c	C 1 2/c 1
Hall group	-C 2yc	-C 2yc
Moiety formula	C28 H16 N8, C4 H10 O, 3(O)	C28 H16 N8, C4 H10 O, 3[H2O]
Sum formula	C32 H26 N8 O4	C32 H26 N8 O4
Mr	586.61	586.61
Dx,g cm-3	1.160	1.160
Z	4	4
Mu (mm-1)	0.654	0.654
F000	1224.0	1224.0
F000'	1227.81	
h,k,lmax	19,15,19	19,15,19
Nref	3071	3060
Tmin,Tmax		
Tmin'		

Correction method= Not given

Data completeness= 0.996 Theta(max)= 68.150

R(reflections)= 0.0907(1642) wR2(reflections)= 0.2975(3060)

S = 0.970 Npar= 230

The following ALERTS were generated. Each ALERT has the format
test-name_ALERT_alert-type_alert-level.
Click on the hyperlinks for more details of the test.

Alert level B

```
PLAT260_ALERT_2_B Large Average Ueq of Residue Including       O1A      0.551 Check
PLAT934_ALERT_3_B Number of (Iobs-Icalc)/Sigma(W) > 10 Outliers ..          2 Check
                0  2  0,  -2  0  2,
```

Alert level C

```
DIFMX02_ALERT_1_C  The maximum difference density is > 0.1*ZMAX*0.75
            The relevant atom site should be identified.
PLAT042_ALERT_1_C Calc. and Reported MoietyFormula Strings  Differ     Please Check
              Calc: C28 H16 N8, C4 H10 O, 3(O)
              Rep.: C28 H16 N8, C4 H10 O, 3[H2O]
PLAT052_ALERT_1_C Info on Absorption Correction Method   Not Given     Please Do !
PLAT053_ALERT_1_C Minimum Crystal Dimension Missing (or Error) ...     Please Check
PLAT054_ALERT_1_C Medium  Crystal Dimension Missing (or Error) ...     Please Check
PLAT055_ALERT_1_C Maximum Crystal Dimension Missing (or Error) ...     Please Check
PLAT084_ALERT_3_C High wR2 Value (i.e. > 0.25) ..................       0.30 Report
PLAT094_ALERT_2_C Ratio of Maximum / Minimum Residual Density ....       2.18 Report
PLAT097_ALERT_2_C Large Reported Max.  (Positive) Residual Density       0.69 eA-3
PLAT260_ALERT_2_C Large Average Ueq of Residue Including      O00K      0.105 Check
PLAT260_ALERT_2_C Large Average Ueq of Residue Including        O1      0.179 Check
PLAT340_ALERT_3_C Low Bond Precision on  C-C Bonds ..............     0.0054 Ang.
PLAT906_ALERT_3_C Large K Value in the Analysis of Variance ......     16.544 Check
PLAT906_ALERT_3_C Large K Value in the Analysis of Variance ......      3.375 Check
PLAT911_ALERT_3_C Missing FCF Refl Between Thmin & STh/L=    0.600          7 Report
                3  1  2,   6  0  8,  10  0  8, -18  4  9,   9  7 10, -12  0 18,
               -3  1 19,
PLAT918_ALERT_3_C Reflection(s) with I(obs) much Smaller I(calc) .          1 Check
```

Alert level G

```
FORMU01_ALERT_1_G  There is a discrepancy between the atom counts in the
            _chemical_formula_sum and _chemical_formula_moiety. This is
            usually due to the moiety formula being in the wrong format.
            Atom count from _chemical_formula_sum:   C32 H26 N8 O4
            Atom count from _chemical_formula_moiety:C32 H32 N8 O4
PLAT007_ALERT_5_G Number of Unrefined Donor-H Atoms .............          3 Report
              H001  H002  H004
PLAT072_ALERT_2_G SHELXL First  Parameter in WGHT  Unusually Large       0.20 Report
PLAT299_ALERT_4_G Atom Site Occupancy Constrained at ............         0.5 Check
              H001    H002    O00K    C1      C2      C3      C4      H1A
              H1B     H2A     H2B     H3A     H3B     H3C     H4A     H4B
              H4C
PLAT302_ALERT_4_G Anion/Solvent/Minor-Residue Disorder (Resd    2)       100% Note
PLAT302_ALERT_4_G Anion/Solvent/Minor-Residue Disorder (Resd    3)       100% Note
PLAT302_ALERT_4_G Anion/Solvent/Minor-Residue Disorder (Resd    4)       100% Note
PLAT304_ALERT_4_G Non-Integer Number of Atoms in ..... (Resd    2)       7.50 Check
PLAT304_ALERT_4_G Non-Integer Number of Atoms in ..... (Resd    3)       0.80 Check
PLAT304_ALERT_4_G Non-Integer Number of Atoms in ..... (Resd    4)       0.70 Check
PLAT311_ALERT_2_G Isolated Disordered Oxygen Atom (No H's ?) .....         O1 Check
```

```
PLAT311_ALERT_2_G Isolated Disordered Oxygen Atom (No H's ?) .....        O1A Check
PLAT720_ALERT_4_G Number of Unusual/Non-Standard Labels ..........         28 Note
              N001    H001    N002    H002    N003    N004    H004    C005
              C006    H006    C007    C008    C009    C00A    C00B    H00B
              C00C    C00D    H00D    C00E    C00F    H00F    C00G    C00H
              H00H    C00I    H00I    O00K
PLAT860_ALERT_3_G Number of Least-Squares Restraints ............          1 Note
PLAT912_ALERT_4_G Missing # of FCF Reflections Above STh/L=  0.600         4 Note
PLAT969_ALERT_5_G The 'Henn et al.' R-Factor-gap value ..........      4.018 Note
              Predicted wR2: Based on SigI**2  7.40 or SHELX Weight 31.16
PLAT978_ALERT_2_G Number C-C Bonds with Positive Residual Density.          0 Info
```

```
   0 ALERT level A = Most likely a serious problem - resolve or explain
   2 ALERT level B = A potentially serious problem, consider carefully
  16 ALERT level C = Check. Ensure it is not caused by an omission or oversight
  17 ALERT level G = General information/check it is not something unexpected

   7 ALERT type 1 CIF construction/syntax error, inconsistent or missing data
   9 ALERT type 2 Indicator that the structure model may be wrong or deficient
   8 ALERT type 3 Indicator that the structure quality may be low
   9 ALERT type 4 Improvement, methodology, query or suggestion
   2 ALERT type 5 Informative message, check
```

It is advisable to attempt to resolve as many as possible of the alerts in all categories. Often the minor alerts point to easily fixed oversights, errors and omissions in your CIF or refinement strategy, so attention to these fine details can be worthwhile. In order to resolve some of the more serious problems it may be necessary to carry out additional measurements or structure refinements. However, the purpose of your study may justify the reported deviations and the more serious of these should normally be commented upon in the discussion or experimental section of a paper or in the "special_details" fields of the CIF. checkCIF was carefully designed to identify outliers and unusual parameters, but every test has its limitations and alerts that are not important in a particular case may appear. Conversely, the absence of alerts does not guarantee there are no aspects of the results needing attention. It is up to the individual to critically assess their own results and, if necessary, seek expert advice.

Publication of your CIF in IUCr journals

A basic structural check has been run on your CIF. These basic checks will be run on all CIFs submitted for publication in IUCr journals (*Acta Crystallographica*, *Journal of Applied Crystallography*, *Journal of Synchrotron Radiation*); however, if you intend to submit to *Acta Crystallographica Section C* or *E* or *IUCrData*, you should make sure that full publication checks are run on the final version of your CIF prior to submission.

Publication of your CIF in other journals

Please refer to the *Notes for Authors* of the relevant journal for any special instructions relating to CIF submission.

PLATON version of 04/06/2025; check.def file version of 30/05/2025

Datablock shelxt - ellipsoid plot

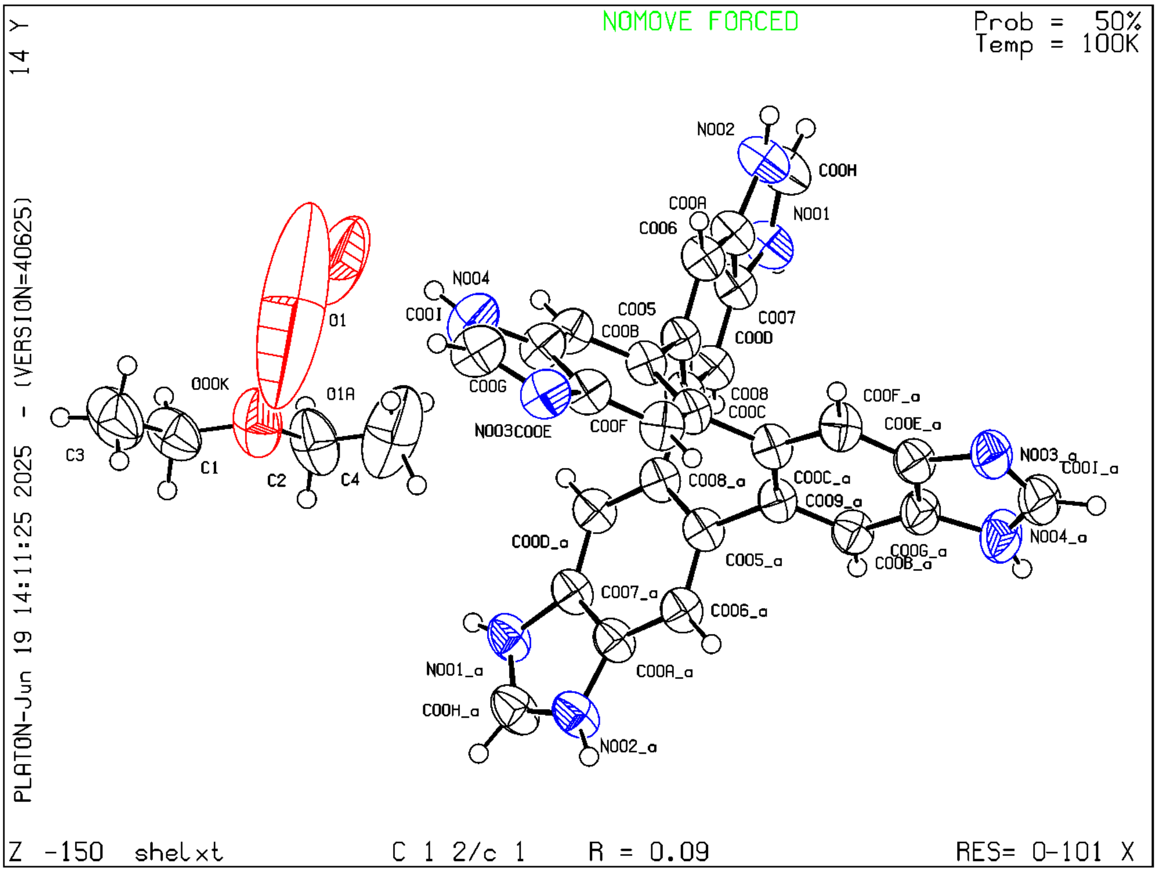

checkCIF/PLATON report

Structure factors have been supplied for datablock(s) shelxt

THIS REPORT IS FOR GUIDANCE ONLY. IF USED AS PART OF A REVIEW PROCEDURE FOR PUBLICATION, IT SHOULD NOT REPLACE THE EXPERTISE OF AN EXPERIENCED CRYSTALLOGRAPHIC REFEREE.

No syntax errors found. CIF dictionary Interpreting this report

Datablock: shelxt

Bond precision:	C-C = 0.0081 A		Wavelength=1.54187
Cell:	a=15.1319(8)	b=13.1893(6)	c=16.3571(8)
	alpha=90	beta=102.978(7)	gamma=90
Temperature:	100 K		

	Calculated	Reported
Volume	3181.2(3)	3181.1(3)
Space group	C 2/c	C 1 2/c 1
Hall group	-C 2yc	-C 2yc
Moiety formula	C28 H17 N8, 2(C H Cl3), Cl	Cl, 2(C H Cl3), C28 H17 N8
Sum formula	C30 H19 Cl7 N8	C30 H19 Cl7 N8
Mr	739.68	739.68
Dx,g cm-3	1.544	1.544
Z	4	4
Mu (mm-1)	6.007	6.007
F000	1496.0	1496.0
F000'	1509.25	
h,k,lmax	18,15,19	18,15,19
Nref	2907	2887
Tmin,Tmax		
Tmin'		

Correction method= Not given

Data completeness= 0.993 Theta(max)= 68.186

R(reflections)= 0.0854(1512) wR2(reflections)= 0.2428(2887)

S = 1.200 Npar= 204

The following ALERTS were generated. Each ALERT has the format
test-name_ALERT_alert-type_alert-level.
Click on the hyperlinks for more details of the test.

Alert level C

```
PLAT042_ALERT_1_C Calc. and Reported MoietyFormula Strings  Differ     Please Check
              Calc: C28 H17 N8, 2(C H Cl3), Cl
              Rep.: Cl, 2(C H Cl3), C28 H17 N8
PLAT052_ALERT_1_C Info on Absorption Correction Method   Not Given     Please Do !
PLAT053_ALERT_1_C Minimum Crystal Dimension Missing (or Error) ...     Please Check
PLAT054_ALERT_1_C Medium  Crystal Dimension Missing (or Error) ...     Please Check
PLAT055_ALERT_1_C Maximum Crystal Dimension Missing (or Error) ...     Please Check
PLAT243_ALERT_4_C High   'Solvent' Ueq as Compared to Neighbors of         C2 Check
PLAT260_ALERT_2_C Large Average Ueq of Residue Including      Cl02      0.144 Check
PLAT340_ALERT_3_C Low Bond Precision on  C-C Bonds ..............    0.00813 Ang.
PLAT906_ALERT_3_C Large K Value in the Analysis of Variance ......     13.957 Check
PLAT906_ALERT_3_C Large K Value in the Analysis of Variance ......      2.974 Check
PLAT911_ALERT_3_C Missing FCF Refl Between Thmin & STh/L=    0.600         13 Report
                8  2 15,   2  0 16,   4  0 16,   6  0 16,   5  1 17,  -2  6 18,
               -1  1 18,   0  0 18,   1  1 18,   2  0 18,   3  1 18,  -2  2 19,
               -1  1 19,
```

Alert level G

```
PLAT007_ALERT_5_G Number of Unrefined Donor-H Atoms .............           4 Report
              H005  H006  H007  H008
PLAT299_ALERT_4_G Atom Site Occupancy Constrained at ............         0.5 Check
              H005    H007    H008
PLAT304_ALERT_4_G Non-Integer Number of Atoms in ..... (Resd    3)       0.50 Check
PLAT344_ALERT_2_G Unusual sp3       Angle Range in Solvent/Ion for         C2 Check
PLAT434_ALERT_2_G Short Inter HL..HL Contact Cl01     ..Cl02     .       3.38 Ang.
                                                       x,y,z  =      1_555 Check
PLAT434_ALERT_2_G Short Inter HL..HL Contact Cl01     ..Cl02     .       3.38 Ang.
                                                 1-x,y,1/2-z  =      2_655 Check
PLAT720_ALERT_4_G Number of Unusual/Non-Standard Labels .........          32 Note
              Cl01    Cl02    Cl03    Cl00    N005    H005    N006    H006
              N007    H007    N008    H008    C009    H009    C00A    C00B
              C00C    H00C    C00D    C00E    C00F    H00F    C00G    C00H
              C00I    H00I    C00J    C00K    C00L    H00L    C00M    H00M
PLAT912_ALERT_4_G Missing # of FCF Reflections Above STh/L=  0.600           7 Note
PLAT969_ALERT_5_G The 'Henn et al.' R-Factor-gap value ...........      4.043 Note
              Predicted wR2: Based on SigI**2  6.00 or SHELX Weight 20.22
PLAT978_ALERT_2_G Number C-C Bonds with Positive Residual Density.           0 Info
```

0 **ALERT level A** = Most likely a serious problem - resolve or explain
0 **ALERT level B** = A potentially serious problem, consider carefully
11 **ALERT level C** = Check. Ensure it is not caused by an omission or oversight
10 **ALERT level G** = General information/check it is not something unexpected

5 ALERT type 1 CIF construction/syntax error, inconsistent or missing data
5 ALERT type 2 Indicator that the structure model may be wrong or deficient
4 ALERT type 3 Indicator that the structure quality may be low
5 ALERT type 4 Improvement, methodology, query or suggestion
2 ALERT type 5 Informative message, check

It is advisable to attempt to resolve as many as possible of the alerts in all categories. Often the minor alerts point to easily fixed oversights, errors and omissions in your CIF or refinement strategy, so attention to these fine details can be worthwhile. In order to resolve some of the more serious problems it may be necessary to carry out additional measurements or structure refinements. However, the purpose of your study may justify the reported deviations and the more serious of these should normally be commented upon in the discussion or experimental section of a paper or in the "special_details" fields of the CIF. checkCIF was carefully designed to identify outliers and unusual parameters, but every test has its limitations and alerts that are not important in a particular case may appear. Conversely, the absence of alerts does not guarantee there are no aspects of the results needing attention. It is up to the individual to critically assess their own results and, if necessary, seek expert advice.

Publication of your CIF in IUCr journals

A basic structural check has been run on your CIF. These basic checks will be run on all CIFs submitted for publication in IUCr journals (*Acta Crystallographica*, *Journal of Applied Crystallography*, *Journal of Synchrotron Radiation*); however, if you intend to submit to *Acta Crystallographica Section C* or *E* or *IUCrData*, you should make sure that full publication checks are run on the final version of your CIF prior to submission.

Publication of your CIF in other journals

Please refer to the *Notes for Authors* of the relevant journal for any special instructions relating to CIF submission.

PLATON version of 04/06/2025; check.def file version of 30/05/2025

Datablock shelxt - ellipsoid plot

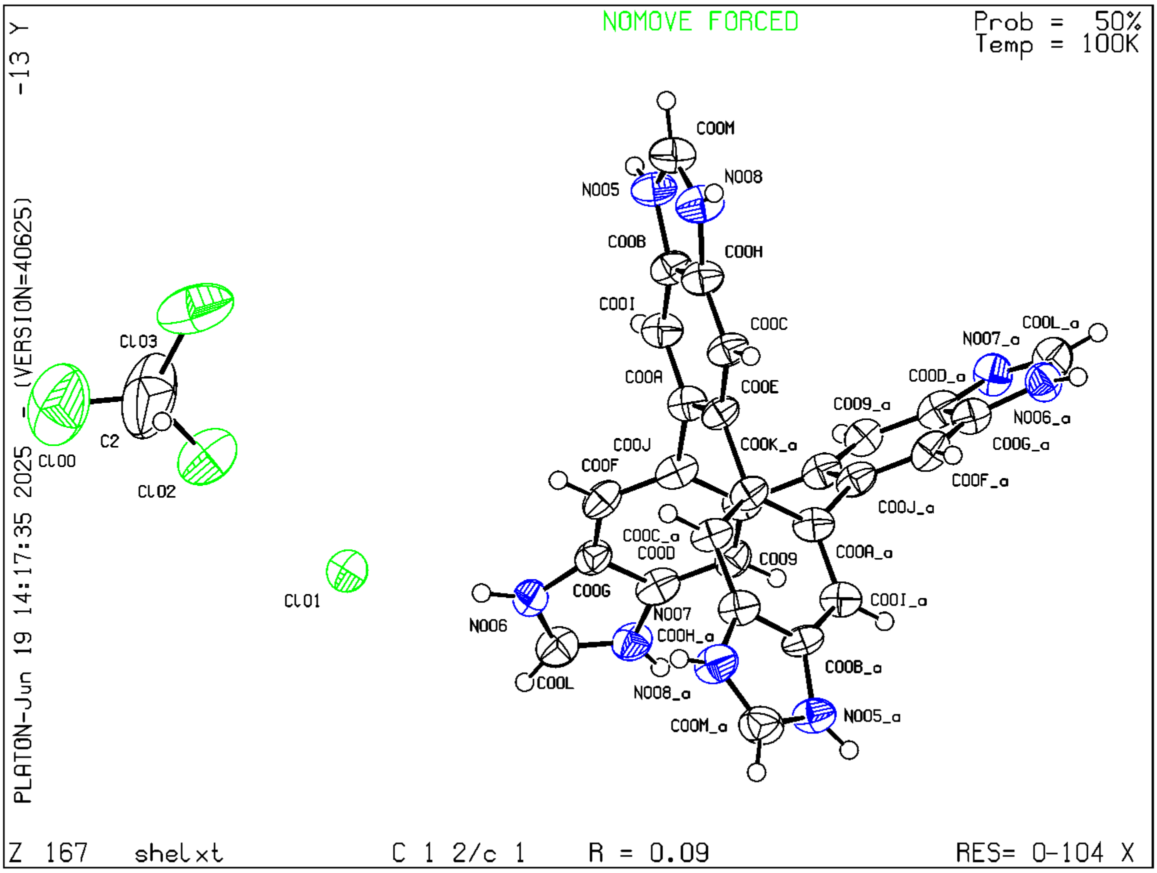

checkCIF/PLATON report

Structure factors have been supplied for datablock(s) shelxt

THIS REPORT IS FOR GUIDANCE ONLY. IF USED AS PART OF A REVIEW PROCEDURE FOR PUBLICATION, IT SHOULD NOT REPLACE THE EXPERTISE OF AN EXPERIENCED CRYSTALLOGRAPHIC REFEREE.

No syntax errors found. CIF dictionary Interpreting this report

Datablock: shelxt

Bond precision:	C-C = 0.0129 A		Wavelength=1.54187
Cell:	a=12.3282(19)	b=12.3282(19)	c=28.108(2)
	alpha=90	beta=90	gamma=90
Temperature:	100 K		

	Calculated	Reported
Volume	4272.0(14)	4272.0(14)
Space group	I -4 2 d	I -4 2 d
Hall group	I -4 2bw	I -4 2bw
Moiety formula	C28 H18 N8, 2(Cl) [+ solvent]	C28 H18 N8, Cl2, 4[C4H8O]
Sum formula	C28 H18 Cl2 N8 [+ solvent]	C44 H50 Cl2 N8 O4
Mr	537.40	825.82
Dx,g cm-3	0.836	1.284
Z	4	4
Mu (mm-1)	1.535	1.785
F000	1104.0	1744.0
F000'	1109.70	
h,k,lmax	14,14,33	14,14,33
Nref	1941[1090]	1941
Tmin,Tmax		
Tmin'		

Correction method= Not given

Data completeness= 1.78/1.00 Theta(max)= 67.811

R(reflections)= 0.0839(674) wR2(reflections)= 0.2254(1941)

S = 0.934 Npar= 91

The following ALERTS were generated. Each ALERT has the format
test-name_ALERT_alert-type_alert-level.
Click on the hyperlinks for more details of the test.

Alert level B

```
PLAT026_ALERT_3_B Ratio Observed / Unique Reflections (too) Low ..         35% Check
PLAT230_ALERT_2_B Hirshfeld Test Diff for    C005     --C008     .         9.2 s.u.
PLAT340_ALERT_3_B Low Bond Precision on  C-C Bonds ...............     0.01286 Ang.
```

Alert level C

```
RINTA01_ALERT_3_C  The value of Rint is greater than 0.12
            Rint given   0.133
PLAT020_ALERT_3_C The Value of Rint is Greater Than 0.12 .........       0.133 Report
PLAT052_ALERT_1_C Info on Absorption Correction Method   Not Given     Please Do !
PLAT053_ALERT_1_C Minimum Crystal Dimension Missing (or Error) ...     Please Check
PLAT054_ALERT_1_C Medium  Crystal Dimension Missing (or Error) ...     Please Check
PLAT055_ALERT_1_C Maximum Crystal Dimension Missing (or Error) ...     Please Check
PLAT234_ALERT_4_C Large Hirshfeld Difference N002     --C007     .        0.20 Ang.
PLAT234_ALERT_4_C Large Hirshfeld Difference N003     --C007     .        0.22 Ang.
PLAT234_ALERT_4_C Large Hirshfeld Difference C004     --C008     .        0.17 Ang.
PLAT234_ALERT_4_C Large Hirshfeld Difference C005     --C009     .        0.17 Ang.
PLAT234_ALERT_4_C Large Hirshfeld Difference C005     --C009_c   .        0.16 Ang.
PLAT234_ALERT_4_C Large Hirshfeld Difference C009     --C005_a   .        0.16 Ang.
PLAT250_ALERT_2_C Large U3/U1 Ratio for <U(i,j)> Tensor(Resd    1)         2.1 Note
PLAT260_ALERT_2_C Large Average Ueq of Residue Including       Cl1       0.136 Check
PLAT905_ALERT_3_C Negative K value in the Analysis of Variance ...      -7.267 Report
PLAT918_ALERT_3_C Reflection(s) with I(obs) much Smaller I(calc) .           3 Check
```

Alert level G

```
FORMU01_ALERT_2_G  There is a discrepancy between the atom counts in the
            _chemical_formula_sum and the formula from the _atom_site* data.
            Atom count from _chemical_formula_sum:C44 H50 Cl2 N8 O4
            Atom count from the _atom_site data:  C28 H18 Cl2 N8
CELLZ01_ALERT_1_G Difference between formula and atom_site contents detected.
CELLZ01_ALERT_1_G ALERT: Large difference may be due to a
            symmetry error - see SYMMG tests
           From the CIF: _cell_formula_units_Z    4
           From the CIF: _chemical_formula_sum  C44 H50 Cl2 N8 O4
           TEST: Compare cell contents of formula and atom_site data

           atom    Z*formula  cif sites diff
           C        176.00    112.00   64.00
           H        200.00     72.00  128.00
           Cl         8.00      8.00    0.00
           N         32.00     32.00    0.00
           O         16.00      0.00   16.00
PLAT007_ALERT_5_G Number of Unrefined Donor-H Atoms .............           2 Report
              H002  H003
PLAT041_ALERT_1_G Calc. and Reported SumFormula    Strings  Differ     Please Check
              Calc: C28 H18 Cl2 N8
              Rep.: C44 H50 Cl2 N8 O4
PLAT042_ALERT_1_G Calc. and Reported MoietyFormula Strings  Differ     Please Check
```

```
              Calc: C28 H18 N8, 2(Cl)
              Rep.: C28 H18 N8, Cl2, 4[C4H8O]
PLAT051_ALERT_1_G Mu(calc) and Mu(cif) Ratio Differs from 1.0 by .      14.01 %
PLAT299_ALERT_4_G Atom Site Occupancy Constrained at ............         0.5 Check
              H003    Cl1
PLAT302_ALERT_4_G Anion/Solvent/Minor-Residue Disorder (Resd    2)       100% Note
PLAT304_ALERT_4_G Non-Integer Number of Atoms in ..... (Resd    2)       0.50 Check
PLAT605_ALERT_4_G Largest Solvent Accessible VOID in the Structure       2179 A**3
PLAT720_ALERT_4_G Number of Unusual/Non-Standard Labels .........          14 Note
              N002    H002    N003    H003    C004    C005    C006    C007
              H007    C008    H008    C009    C00A    H00A
PLAT868_ALERT_4_G ALERTS Due to the Use of _smtbx_masks Suppressed          ! Info
PLAT916_ALERT_2_G Hooft y and Flack x Parameter Values Differ by .       0.11 Check
PLAT969_ALERT_5_G The 'Henn et al.' R-Factor-gap value ..........       4.782 Note
              Predicted wR2: Based on SigI**2  4.71 or SHELX Weight 24.14
PLAT978_ALERT_2_G Number C-C Bonds with Positive Residual Density.          0 Info
```

---

0 **ALERT level A** = Most likely a serious problem - resolve or explain
3 **ALERT level B** = A potentially serious problem, consider carefully
16 **ALERT level C** = Check. Ensure it is not caused by an omission or oversight
16 **ALERT level G** = General information/check it is not something unexpected

9 ALERT type 1 CIF construction/syntax error, inconsistent or missing data
6 ALERT type 2 Indicator that the structure model may be wrong or deficient
6 ALERT type 3 Indicator that the structure quality may be low
12 ALERT type 4 Improvement, methodology, query or suggestion
2 ALERT type 5 Informative message, check

It is advisable to attempt to resolve as many as possible of the alerts in all categories. Often the minor alerts point to easily fixed oversights, errors and omissions in your CIF or refinement strategy, so attention to these fine details can be worthwhile. In order to resolve some of the more serious problems it may be necessary to carry out additional measurements or structure refinements. However, the purpose of your study may justify the reported deviations and the more serious of these should normally be commented upon in the discussion or experimental section of a paper or in the "special_details" fields of the CIF. checkCIF was carefully designed to identify outliers and unusual parameters, but every test has its limitations and alerts that are not important in a particular case may appear. Conversely, the absence of alerts does not guarantee there are no aspects of the results needing attention. It is up to the individual to critically assess their own results and, if necessary, seek expert advice.

Publication of your CIF in IUCr journals

A basic structural check has been run on your CIF. These basic checks will be run on all CIFs submitted for publication in IUCr journals (*Acta Crystallographica*, *Journal of Applied Crystallography*, *Journal of Synchrotron Radiation*); however, if you intend to submit to *Acta Crystallographica Section C* or *E* or *IUCrData*, you should make sure that full publication checks are run on the final version of your CIF prior to submission.

Publication of your CIF in other journals

Please refer to the *Notes for Authors* of the relevant journal for any special instructions relating to CIF submission.

PLATON version of 04/06/2025; check.def file version of 30/05/2025

Datablock shelxt - ellipsoid plot

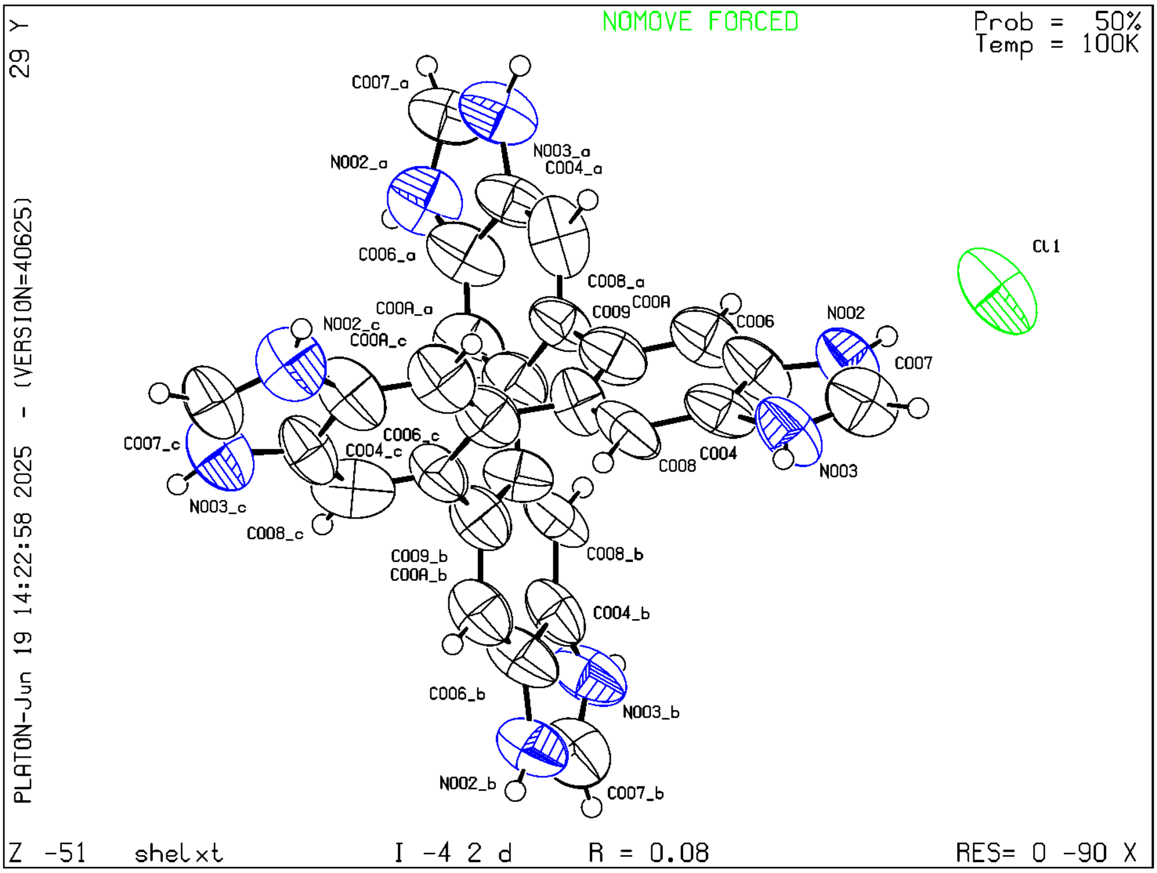

Supplement: Supplementary file 2 — Supporting Information [file CHEM-31-e02622-s001.zip › CheckCIF.pdf]
